# DDCANet for SiO_2_–mediated drought regulation research: high–precision segmentation and phenotypic detection of cucumber point clouds

**DOI:** 10.3389/fpls.2026.1848132

**Published:** 2026-06-10

**Authors:** Yuanyu Xia, Yitong Liu, Sheng Zhu, Siying Liu, Haoqi Wang, Shihan Wang, Yujuan Xu, Ziyi Zhang, Hongbin Wu, Xiuqing Fu

**Affiliations:** College of Engineering, Nanjing Agricultural University, Nanjing, China

**Keywords:** cucumber seedlings, drought stress, Euclidean clustering, semantic segmentation, SiO_2_ nanoparticles

## Abstract

Cucumber is a core cultivated facility vegetable in China. Drought stress at the seedling stage severely inhibits its growth and development. The regulatory mechanism and optimal application concentration of SiO_2_ nanoparticles in alleviating drought stress in cucumber seedlings remain unclear. Moreover, traditional manual measurement and classic point cloud segmentation models struggle to achieve high-throughput accurate detection of cucumber seedling phenotypes under drought stress. To address these issues, this study focused on phenotypic detection under drought stress and analysis of the regulatory effects of SiO_2_ nanoparticles. An improved compact and low-redundancy segmentation model, DDCANet, was proposed based on PointNet++-SSG. Combined with 3D point cloud technology and the Euclidean clustering algorithm, it enables automatic extraction of phenotypic parameters from cucumber seedlings treated with SiO_2_ nanoparticles under drought stress. In this study, Trailing cucumber seedlings were used as experimental materials. 3D point cloud data of cucumber seedlings were collected under treatments with different concentrations of SiO_2_ nanoparticles and PEG-simulated drought stress. A dataset containing 70 valid samples was constructed and labeled into two categories: Stem and Leaf. The core optimizations of the DDCANet model are as follows: Firstly, an Adaptive Density-Aware Feature Enhancement (ADFE) module is embedded to accurately capture point cloud density heterogeneity induced by SiO_2_;Secondly, a Channel Attention and Normalization-enhanced SA Layer (CANL) is designed to strengthen the coupling of local and global drought-related phenotypic features. Thirdly, a Drought-Aware Hybrid Loss (DHL) function is constructed to alleviate the class imbalance of seedling stem and leaf point clouds under drought stress. Results show that the DDCANet model achieves a mean Intersection over Union (mIoU) of 89.01 ± 0.32% and a Stem IoU of 83.6 ± 0.45%, representing improvements of 6.55% and 9.6% respectively compared with the baseline PointNet++-SSG model, and a 30.5% improvement in stem segmentation accuracy compared with the classic PointNet model. It thus enables high-throughput, non-destructive detection of drought phenotypes in cucumber seedlings under SiO_2_ nanoparticle treatment. Ablation experiments verified the positive contributions of the ADFE, CANL, and DHL modules. Furthermore, instance segmentation and phenotype extraction were completed using the Euclidean clustering algorithm to analyze the drought-alleviating effects of SiO_2_ nanoparticles under PEG-simulated drought stress. Results indicate that a low concentration of 20 mg/L exhibits a weak alleviating effect, medium concentrations of 40–60 mg/L show bidirectional regulatory characteristics, and a high concentration of 100 mg/L causes negative physiological effects. The optimal application concentration is 80 mg/L, which comprehensively improves key phenotypes such as seedling height and volume under drought stress and exerts a positive regulatory effect on seedling growth under drought conditions. The DDCANet model constructed in this study provides an efficient technical tool for the accurate phenotypic detection of crop seedlings treated with SiO_2_ nanoparticles under drought stress. It clarifies the optimal application concentration of SiO_2_ nanoparticles, offers a precise concentration threshold and theoretical support for the scientific application of SiO_2_ nanoparticles in drought-stressed cultivation of protected cucumber, and establishes a novel methodological reference for the research on phenotypic regulation of crops under drought stress via nano-agricultural technology.

## 1 Introduction

Cucumber is a core cultivated crop in the protected vegetable cultivation system of China, with extremely high economic value and market demand. The growth and development status at the seedling stage directly determines the stress resistance, yield formation, and fruit quality of the plant throughout the growth period. With the intensification of global climate change, drought stress has become a major abiotic stress factor restricting the high-quality and efficient production of protected cucumber in China. As the most sensitive growth stage to drought stress, water deficit at the seedling stage significantly inhibits the morphogenesis of seedling roots, stems and leaves, disrupts the structural stability of chloroplasts and photosynthetic system, induces cellular oxidative damage, and ultimately leads to a substantial decline in cucumber yield and quality (El-Mogy et al., 2025). Therefore, exploring efficient, safe and environmentally friendly drought-resistant regulation technologies for cucumber seedlings, and establishing accurate, high-throughput and non-destructive dynamic monitoring methods for seedling growth phenotypes have become key scientific and technical issues urgently to be solved in the fields of stress-resistant cultivation and genetic breeding of protected cucumber.

In recent years, the rapid development of nano-agricultural technology has provided a new approach for the green regulation of crop abiotic stresses. Silica nanoparticles (SiO_2_ NPs) exhibit great application potential in alleviating crop abiotic stresses owing to their unique small-size effect, high surface activity, excellent biocompatibility and environmental safety. Nanoparticles have been widely recognized as ideal carriers for delivering bioactive functional substances in agriculture, which provides theoretical support for the foliar and seed priming application mode of SiO2 NPs in this study (Ma, 2026). Existing studies have confirmed that SiO_2_ NPs can enhance crop drought tolerance through synergistic regulation in multiple ways, including forming a physical barrier on the leaf surface to reduce transpiration water loss, protecting the structural and functional stability of photosynthetic organs, activating the plant antioxidant defense system, regulating cellular osmotic balance and the endogenous hormone signaling network, etc (Daler et al., 2025). Studies on crops such as grape and wheat have clarified the concentration-dependent effect of SiO_2_ NPs on drought resistance regulation: foliar spraying of 10 ppm significantly alleviates the growth inhibition of grape seedlings caused by drought, and 60 ppm treatment achieves the optimal improvement effect on wheat drought resistance (Boora et al., 2023a). Preliminary studies on cucumber have also found that seed priming with 40 mg/L SiO_2_ NPs significantly improves the germination rate and seedling vigor of cucumber seeds under simulated drought conditions, and the induced stress resistance has transgenerational genetic potential. However, the systematic regulatory effect of SiO_2_ NPs on drought tolerance of cucumber seedlings remains to be further analyzed, and the dose-response pattern of different concentrations, physiological and biochemical regulation mechanisms, as well as their effects on the three-dimensional morphogenesis of stems and leaves of cucumber seedlings under drought stress, still lack accurate quantitative characterization and systematic elucidation.

Plant phenomics is a key bridge linking crop genotypes, environmental factors and stress-resistant phenotypes, and accurate and efficient extraction of phenotypic parameters is the core prerequisite for analyzing the crop drought response mechanism (Benallal et al., 2025). With the development of 3D computer vision, real-time semantic understanding of point clouds has become a new research hotspot, which further expands the application potential of point cloud technology in plant phenotyping (Hou et al., 2025). Traditional phenotypic measurement of cucumber seedlings mainly relies on manual measurement and two-dimensional image analysis, which has significant technical limitations: manual measurement is subjective, poor in data consistency and low in efficiency, failing to meet the requirements of large-scale and high-throughput phenotypic collection; two-dimensional image analysis cannot fully capture the three-dimensional spatial structure information of plants, making it difficult to accurately quantify key phenotypes such as leaf inclination, canopy volume and stem-leaf spatial distribution; meanwhile, traditional detection methods for most physiological indicators require destructive sampling, which cannot realize continuous dynamic monitoring of the same plant. The emergence of three-dimensional (3D) point cloud technology provides a revolutionary solution for the non-destructive and accurate acquisition of plant 3D phenotypes, and has been successfully applied in various scenarios from satellite remote sensing to indoor plant monitoring (Jawak et al., 2023). Through non-contact methods such as laser scanning and structured light, it can completely record the precise coordinate information of plants in 3D space, laying a data foundation for crop organ segmentation and automated extraction of phenotypic parameters (Liu et al., 2026).

With the rapid development of deep learning technology in the field of 3D point cloud processing, deep learning-based point cloud semantic segmentation has become the mainstream technical direction for crop 3D phenotype analysis. A large number of improved deep learning detection architectures have been proposed in recent years, laying a solid algorithm foundation for plant phenotypic feature extraction (Yang et al., 2023). According to different data processing methods, relevant methods can be divided into four categories: point-based, projection-based, voxel-based and multimodal fusion methods. Among them, point-based methods represented by PointNet++ have been most widely used in plant organ point cloud segmentation due to their advantages of directly processing raw point cloud data and retaining the original geometric information of plants to the greatest extent (Benallal et al., 2025). Multiple comparative studies have confirmed that PointNet++ achieves significantly higher accuracy than other point-based deep learning architectures such as PointNet and DGCNN in the point cloud semantic segmentation task of crops including rose bushes. Its core advantage lies in the hierarchical learning architecture that can flexibly adapt to the structural changes of plant organs at different scales (Turgut et al., 2022). Nevertheless, for the complex structure of cucumber seedlings with slender stems and thin leaves interlaced and overlapped, traditional PointNet++ still has problems such as insufficient integration of global context information, low precision in capturing organ edge features, and poor adaptability to organ size changes at different growth stages. The PointSegNet proposed by Qiao et al. (Qiao et al., 2025) has achieved 93.73% mean Intersection over Union (mIoU) in stem-leaf segmentation of crops such as corn and tomato by introducing a global-local set abstraction module and an edge-aware feature propagation module, providing an important reference for the optimization of PointNet++. However, compact and low-redundancy segmentation models specially optimized for the morphological characteristics of cucumber seedlings are still scarce, and the balance between segmentation accuracy, generalization performance and computational efficiency of existing models needs to be further improved.

Based on the above research background and existing problems, this study takes “Trailing” cucumber seedlings as the research object and carries out two core studies: First, on the basis of the classic PointNet++-SSG encoder-decoder architecture, embed the Adaptive Density Feature Enhancement (ADFE) module, Channel Attention and Normalization Layer (CANL) branch, and design an adaptive composite loss function (DHL) fusing weighted cross-entropy and Dice loss, to construct a compact and low-redundancy point cloud semantic segmentation model DDCANet for stem-leaf segmentation of drought-stressed cucumber seedlings, realizing high-precision automatic segmentation of stem and leaf organs of cucumber seedlings. Innovatively introduce the Euclidean clustering algorithm for instance segmentation to complete the automated extraction of key 3D phenotypic parameters such as plant height, volume, compactness and X- and Y-direction crown width. Second, set up SiO_2_ nanoparticle priming treatments with gradient concentrations of 0, 20, 40, 60, 80 and 100 mg/L, combined with 60 g/L PEG solution to simulate drought stress environment, systematically analyze the effects of different concentrations of SiO_2_ nanoparticles on the growth dynamics of cucumber seedlings at the seedling stage (36 h to 96 h after sowing), and clarify the concentration effect and time cumulative effect of their drought resistance regulation. This study aims to provide an efficient technical tool for high-throughput, non-destructive and automated extraction of 3D phenotypes of cucumber seedlings, reveal the phenotypic response mechanism of SiO_2_ nanoparticles alleviating drought stress in cucumber seedlings, and supply theoretical basis and technical support for the scientific application of SiO_2_ nanoparticles in drought-resistant cultivation of protected cucumber.

## 2 Materials and methods

### 2.1 Experimental equipment and experimental design

**Figure 1:**
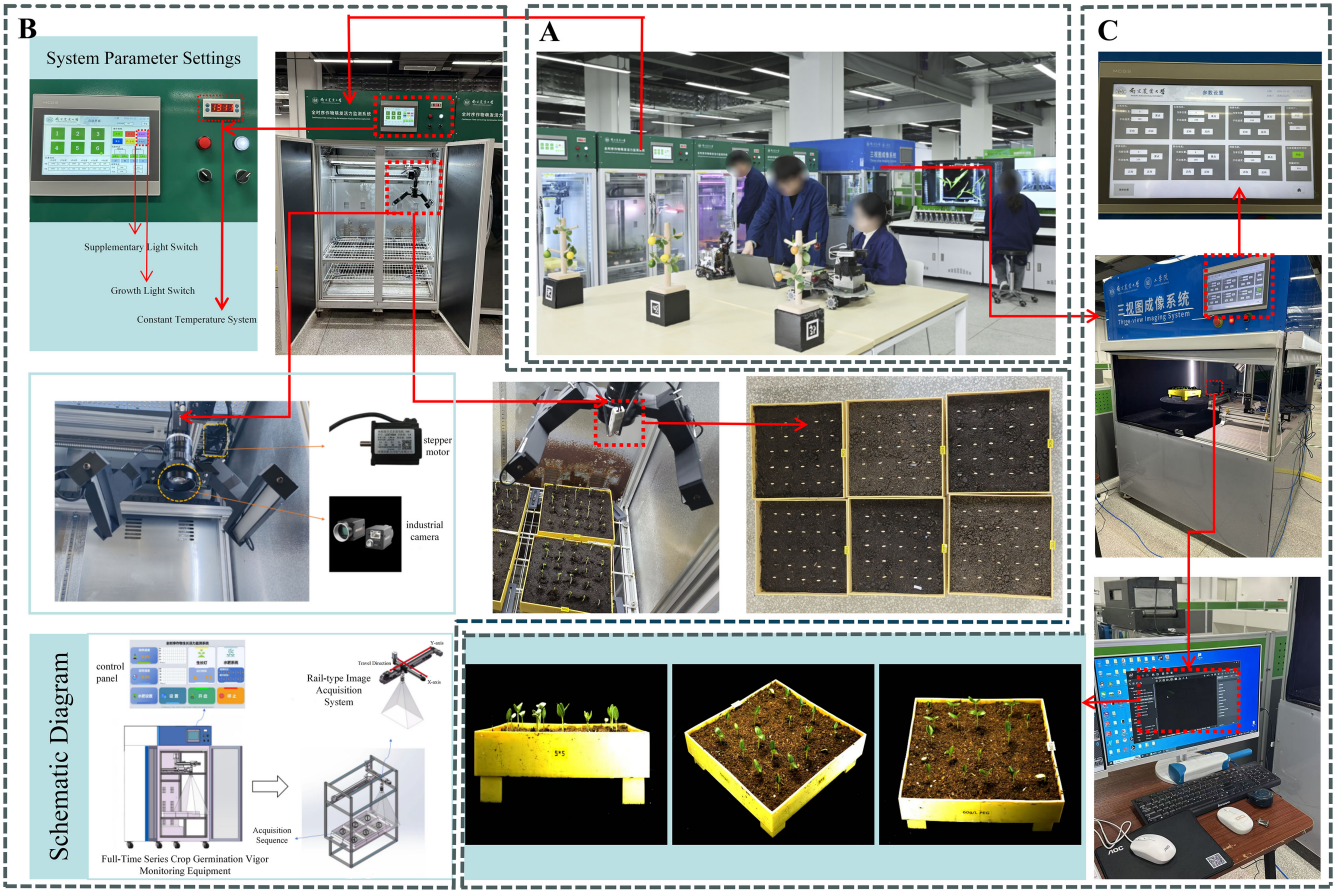
Comprehensive diagram of the experimental device and technical process of the crop phenotypic information acquisition system [**(A)** Actual scene of the experimental operation area for crop phenotypic information collection; **(B)** Crop growth environment control system and rail-type image acquisition module; **(C)** Three-view imaging system and phenotypic data processing terminal module].

As shown in **Figure 1A**, the system consists of a crop growth cultivation system (including a cultivation room environmental control module and a rail-type image acquisition module) and a three-view imaging system (Xie and Yang, 2020).

As shown in **Figure 1B**, in the crop growth cultivation system, users complete the parameter preset and switch control of functions such as supplementary lighting, growth lamps, and constant temperature system through the system parameter setting touch screen. After the instructions are transmitted to the incubator, its built-in temperature control and sensing components automatically adjust indicators such as temperature, humidity, and gas concentration, building a closed automated cultivation environment adapted to crop growth. Crop seeds or seedlings are placed in partitioned cultivation boxes, moved to the bearing area at the lower layer of the incubator for cultivation, and deionized water is sprayed regularly to provide experimental water source. The perforated partition further optimizes the stability of the cultivation environment; the RGB industrial camera (Liang et al., 2024) regularly scans and shoots along the preset track, and the images are transmitted to the industrial computer for storage through Gigabit Ethernet.

As shown in **Figure 1C**, when monitoring is required, the cultivation box is transferred to the three-view imaging system. The ball screw module drives the MV-CS200-10GC color camera to adjust the shooting position, and the electric lifting and rotating table drives the cultivation box to adjust the angle and height, completing the hardware preparation before imaging. During imaging, the MV-CS200-10GC color camera, combined with LED light source for supplementary lighting, collects high-definition images of the crop, capturing the details of growth status in real time. The collected images and growth data are first transmitted to the on-site central control display terminal for preliminary display, and then uploaded to the computer terminal control system for storage and analysis.

**Figure 2:**
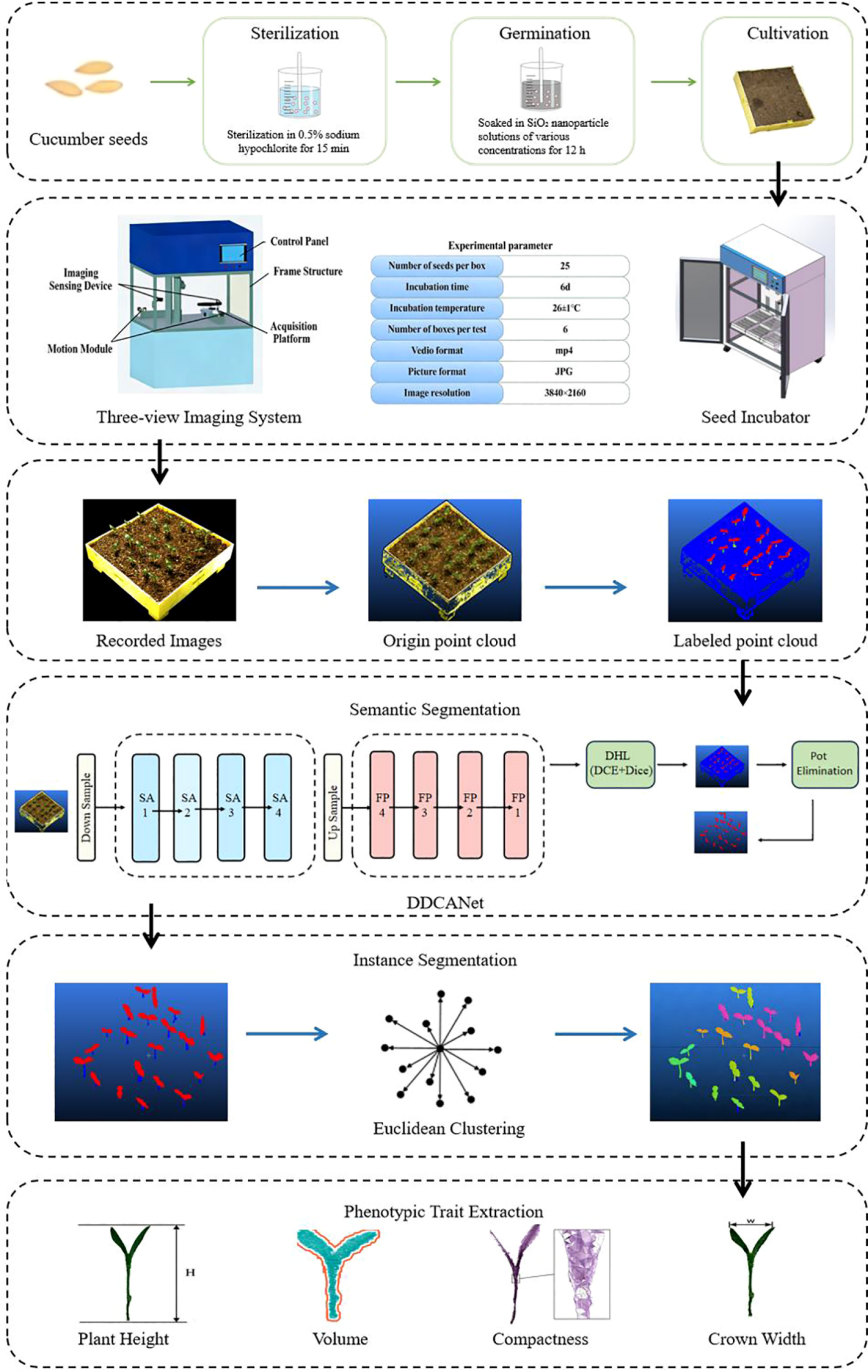
Flowchart of phenotype acquisition for cucumber seedlings.

The structural framework proposed in this paper for acquiring phenotypic information of cucumber seedlings is shown in **Figure 2**:

### 2.2 Experimental materials and dataset construction

Healthy cucumber seeds of the cultivar Trailing with full grains and no disease or pest damage were selected as the experimental material. The seeds were sown in a 25 cm×25 cm×5 cm culture box in a 5×5 grid arrangement. Phenotypic data collection was performed at the critical seedling stage when plant height reached 3–5 cm after emergence. A total of 144 video clips of cucumber seedlings were acquired using image capture devices, and 70 high-quality video sequences were retained after validity screening. Three-dimensional reconstruction was conducted from both front view and axonometric view using COLMAP software for image feature matching, generating 3D point cloud files in. PLY format (Liequan et al., 2024) to realize the digital representation of seedling geometric morphology.

In this study, a single sample in the dataset is clearly defined as the reconstructed 3D point cloud of an individual potted cucumber seedling acquired at a single monitoring time point, with background and other irrelevant redundant point cloud data removed. To achieve organ-level semantic segmentation of point clouds, manual annotation of 3D point clouds was performed using CloudCompare software (Jia-le et al., 2023). Point clouds were initially classified into three categories according to organ attributes: pot, leaf, and stem, labeled with digital tags 0, 1, and 2 respectively. All annotations were independently performed by a researcher with extensive experience in 3D plant point cloud annotation. Clear criteria for distinguishing stem and leaf organs were established prior to annotation, and unified training was conducted to ensure consistency in labeling rules. During the annotation process, previously labeled samples were regularly reviewed to maintain stable annotation standards and reduce subjective bias. Since this study focused on phenotypic analysis of cucumber seedlings (e.g., stem and leaf morphology, spatial distribution), the pot was only a cultivation carrier with no direct relevance to seedling phenotypes, and its point cloud data would increase redundancy and computational cost. Therefore, point clouds corresponding to the pot were removed from the annotated results, retaining only two core organ classes: leaf (label 1) and stem (label 2). The processed point cloud data were then stored in TXT format, where each row represented multi-dimensional features of a single point, including 3D spatial coordinates, organ category labels, and relevant index parameters. This standardized text format enabled structured storage and convenient access for subsequent algorithms (Li X. et al., 2024).

The global point distribution of stem and leaf categories in this dataset is as follows: the stem point cloud contains 441, 044, 440 points, accounting for 77.45% of the total; the leaf point cloud contains 128, 441, 080 points, accounting for 22.55% of the total. The data distribution is consistent with the actual characteristics of plant 3D point clouds, with no severe class imbalance. To improve model robustness and generalization ability and alleviate overfitting caused by limited original sample size, a data augmentation strategy of rotation around the Z-axis was introduced. Random angle rotation was applied to preprocessed point cloud samples, enriching spatial posture diversity and expanding effective training data without altering the 3D geometric structure and semantic labels of seedlings. Finally, taking each culture pot as an independent unit, we evaluated the model performance using 3-fold cross-validation: the dataset was randomly divided into 3 mutually non-overlapping subsets, with 2 subsets used as the training set and 1 subset as the test set in turn. Point cloud data collected at different time points from the same pot were assigned to the same subset to avoid temporal data leakage and ensure reliable evaluation of model generalization. The construction of the 3D phenotypic dataset for cucumber seedlings was thus completed.

### 2.3 Semantic segmentation design based on PointNet++ (DDCANet)

#### 2.3.1 Basic model (PointNet++-SSG)

This study utilized a dataset of 140 cucumber seedlings point cloud samples. Through stratified representative sampling, 70 samples covering all time gradients and stress concentrations were selected for model development, with the remaining samples reserved as an independent external validation set. Considering the dataset characteristics of 70 self-built samples, PointNet++-SSG was finally selected as the basic model for semantic segmentation, and the Multi-Scale Grouping (MSG) scheme was not adopted, because its core architecture matches the experimental requirements perfectly, as similarly demonstrated by Wang et al. (Wang et al., 2023).

As a single-scale grouping variant of PointNet++, PointNet++-SSG mainly consists of an input layer, multi-layer Set Abstraction (SA) modules, Feature Propagation (FP) modules, and an output layer. In the architecture, the input layer receives 3D coordinates and feature information of cucumber seedling point clouds. In the first SA module, Furthest Point Sampling (FPS) is first used to select centroid points from the seedling point clouds, and then Ball Query with a fixed radius is applied to construct single-scale local neighborhoods. Subsequently, the PointNet sub-network extracts and aggregates features within the neighborhood to generate a sparser point cloud feature set. Subsequent SA modules repeat this single-scale feature extraction process to gradually achieve feature abstraction from local to global for seedling point clouds. The FP module maps high-level abstract features back to the original point cloud resolution through interpolation and concatenation operations. Finally, a multi-layer perceptron outputs semantic segmentation results of stems, leaves and other parts of cucumber seedlings (Sarker et al., 2024).

The overall architecture abandons redundant multi-scale computation branches, greatly reducing parameter scale and computational complexity. This compact, concise and efficient design is well-suited for the small-scale data scenario with only 70 self-built samples in this experiment, which can effectively avoid model overfitting. Meanwhile, it accurately captures geometric features and semantic information of key structures such as stems and leaves in cucumber seedling point clouds, meeting the demand for extracting drought tolerance-related traits of seedlings under silica nanoparticle stress.

Although the MSG multi-scale grouping architecture achieves multi-scale feature fusion through multi-radius neighborhood search and feature concatenation, which can theoretically capture local geometric details more finely, this architecture requires multiple grouping scales in the same SA layer with separate feature extraction. The network parameters and training difficulty increase exponentially, and its requirements for sample quantity and data diversity are much higher than the 70 self-built samples in this experiment. It is not only difficult to achieve sufficient training, but also prone to problems such as feature redundancy, slow training convergence and large fluctuations in segmentation accuracy, which cannot meet the practical task objectives of semantic segmentation of cucumber seedling point clouds. Recent studies have attempted to introduce attention mechanisms into PointNet++ to enhance local feature extraction, which provides an important reference for our model optimization (Cheng and Gu, 2024).

The above adaptability analysis of the SSG and MSG approaches is further supported by the comparative segmentation performance results of PointNet++-SSG and PointNet++-MSG presented in Section 2.7, which confirms the rationality of our chosen scheme for the task at hand.

#### 2.3.2 Overall architecture of the improved model

**Figure 3:**
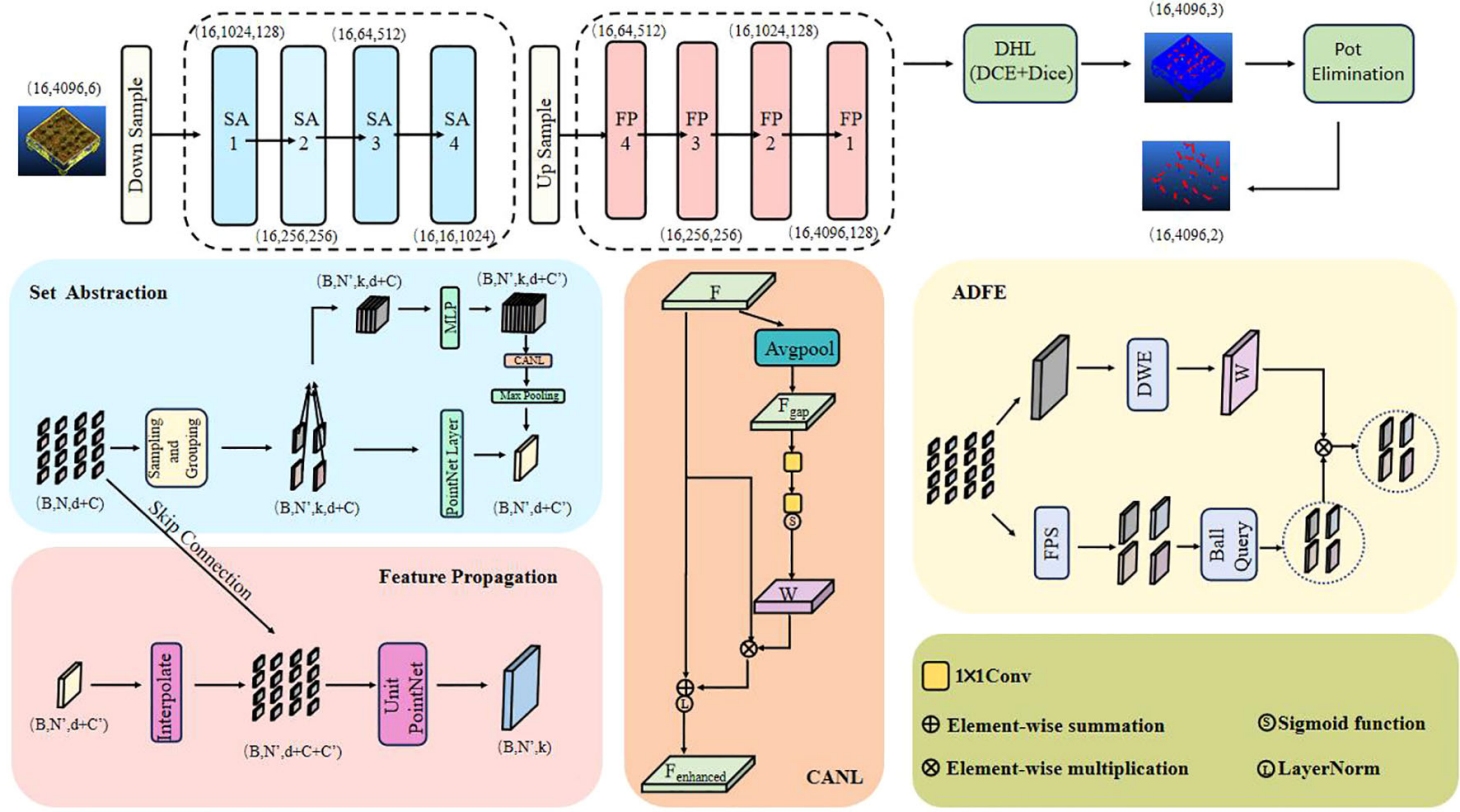
Architecture of DDCANet.

This study proposes DDCANet. On the basis of PointNet++-SSG, density-aware enhancement, channel attention and normalized feature branches are embedded after the SA module (Fei et al., 2023) to realize the collaborative enhancement of local geometric features and global semantic features. Then the final segmentation is completed through FP upsampling and hybrid loss supervision. The overall architecture compact and low-redundancy, stable training and high robustness (Betsas et al., 2025). The specific architecture diagram is shown in **Figure 3**.

#### 2.3.3 Core modules

##### 2.3.3.1 Module 1. Adaptive density feature enhancement for SiO_2_-induced density heterogeneity

Modified treatment with SiO_2_ nanoparticles significantly alters the physiological structure and spatial morphology of cucumber seedlings, directly resulting in heterogeneous point cloud distribution characterized by sparse leaves and dense stems. Point cloud density heterogeneity has been recognized as one of the main challenges limiting the accuracy of semantic segmentation models, and density-aware downsampling methods have been proposed to address this issue (Jiang et al., 2025; Zhou, 2023). Leaf regions show reduced and discrete sampling points due to nanoparticle-induced stomatal regulation and tissue development changes; stem regions exhibit significantly enhanced point aggregation due to increased lignification, forming high-density core areas. The feature extraction pipeline of the traditional PointNet++ model relies only on Euclidean distance metrics of geometric coordinates and is highly sensitive to local density variations. When processing such heterogeneous point clouds, the dominant features in high-density stem regions tend to suppress small-target feature representation in low-density leaf regions, ultimately leading to feature deviation and class imbalance in model recognition of drought stress phenotypes in cucumber seedlings.

To address these issues, this study proposes the Adaptive Density Feature Enhancement (ADFE) module. Based on the physical property of SiO_2_-induced density heterogeneity in cucumber seedling point clouds, this module converts spatial distribution density into interpretable feature weighting coefficients, achieving feature retention in high-density stem regions and feature enhancement in low-density leaf regions, thereby mitigating representation bias caused by density differences at the data level. The module has two core characteristics: First, it requires no additional learnable parameters and realizes density quantification and feature weighting only through tensor operations, maintaining the low-redundancy nature of the model. Second, it is naturally compatible with the single-scale grouping (SSG) structure of PointNet++ and can be directly embedded into the Sampling and Grouping stage of the Set Abstraction (SA) module without large-scale reconstruction of the backbone network. The complete implementation steps of the ADFE module are detailed in Equations 1–8.

(1) Construction principle of the local density descriptor.

The core of the module lies in constructing an interpretable local density descriptor based on k-Nearest Neighbor (k-NN) neighborhood analysis, which converts the spatial distribution density of the point cloud into feature weighting coefficients. For the input coordinate tensor of cucumber seedling point clouds $xyz\in {\mathbb{R}}^{B\times C\times N}$ (where *B* denotes batch size, *C* = 3 represents the 3D coordinate dimension, and *N* is the total number of points of a single seedling), the calculation of local density follows the negative correlation between distance and density: the smaller the average Euclidean distance of the k-nearest neighbors of a point, the higher the aggregation degree of the local point cloud and the larger the density value; otherwise, the density value is smaller.

Step 1: Coordinate tensor dimension transformation.

To match the Euclidean distance calculation logic, first perform dimension permutation on the input coordinate tensor, converting the channel-point arrangement [*B*, *N*, *C*] to point-coordinate arrangement [*B*, *N*, *C*]:

(1) $$xyz_{trans}=permute\left(xyz,\left(0,2,1\right)\right)$$

where *permute*(·) denotes tensor dimension permutation, yielding $xyz_{trans}\in {\mathbb{R}}^{B\times N\times C}$.

Step 2: k-NN Euclidean distance calculation.

Using tensor expansion and broadcasting, compute the Euclidean distance matrix $dist\in {\mathbb{R}}^{B\times N\times N}$ between every pair of points:

(2) $$dist_{b,i,j}=\sqrt{\overset{C}{\underset{c=1}{\sum }}{\left(xyz_{trans,b,i,c}-xyz_{trans,b,j,c}\right)}^{2\;}}$$

where *b* ∈ [1, *B*] is the batch index, *i*, *j* ∈ [1, *N*] are point indices, and *c* ∈ [1, *C*] is the coordinate dimension index. Then use the *topk*(·) function to retrieve the *K* nearest neighbors for each point (In this study, we set *K* = 16. This value was determined based on the point cloud density and local scale characteristics of cucumber seedlings: the point cloud density of individual plants in our dataset ranges from 10^4^ to 10^5^ points, and the local geometric scales of stems and leaves are relatively small. *K* = 16 enables the model to capture sufficient local features while avoiding feature redundancy and excessive computational overhead caused by overly large *K* values. Meanwhile, this configuration refers to the common settings of PointNet++ in similar agricultural point cloud segmentation tasks and is highly adapted to the point cloud density and organ scale characteristics in this study. Therefore, *K* = 16 was finally adopted.), obtaining the k-NN distance tensor $knn\_dist\in {\mathbb{R}}^{B\times N\times K}$:

(3) $$knn_{d}ist=topk\left(dist,k=K,dim=-1,largest=False\right)$$

Step 3: Quantification and normalization of local density values.

Take the mean of *knn*_*dist* along the neighbor dimension (*dim* = −1) to obtain the average k-NN distance $avg\_dist\in {\mathbb{R}}^{B\times N\times 1}$ for each point:

(4) $$avg\_dist_{b,i}=\frac{1}{K}\overset{K}{\underset{k=1}{\sum }}knn\_dist_{b,i,k}$$

Use the reciprocal of the average distance as the initial density value, adding 10^−8^to avoid division by zero:

(5) $$density_{init,b,i}=\frac{1}{avg\_dist_{b,i}+10^{-8}}$$

To eliminate density scale differences among different samples, min–max normalization is performed within the *density_init_* batch to map density values to the interval [0, 1].

(6) $$density_{b,i}=\frac{density_{init,b,i}-\underset{i}{\min}(density_{init,b,i})}{\underset{i}{\max}(density_{init,b,i})-\underset{i}{\min}(density_{init,b,i})+10^{-8}}$$

The normalized $density\in {\mathbb{R}}^{B\times 1\times N}$ accurately characterizes the local *density* distribution of cucumber seedling point clouds: the *density* value approaches 1 in dense stem regions and 0 in sparse leaf regions.

(2) Implementation of density-weighted feature enhancement.

In the FPS sampling stage of the SA module, the indices $fps\_idx\in {\mathbb{R}}^{B\times n_{point}}$ of *n_point_* sampled points are obtained. Based on these indices, the matching and weighting of density weights and feature tensors are completed.

Step 1: Extraction of density weights for sampled points.

Through the *index*_*points*(·) function, the density values corresponding to the sampled points are extracted from the density tensor, and the dimension is expanded to match the dimensions of the feature tensor.

(7) $$group_{d}ensity=unsqueeze\left(index_{p}oints\left(density,fps_{i}dx\right),2\right)$$

Finally, the density weight tensor $group\_density\in {\mathbb{R}}^{B\times n_{point}\times 1\times 1}$ is obtained, where *n_point_* 为denotes the number of sampling points in the SA module.

Step 2: Density weighting of point cloud features.

Multiply the sampled point cloud features $\;new\_points\in {\mathbb{R}}^{B\times n_{point}\times C_{feat}\times N_{sample}}\;$ (where *C_feat_* is the number of feature channels and *N_sample_* is the number of neighborhood sampling points) with the density weights element-wise to achieve adaptive feature enhancement.

(8) $$new\_points_{weighted}=new\_points⊙group\_density$$

where ⊙ denotes the element-wise multiplication operation. This operation preserves the basic feature response of high-density stem regions (with weights close to 1). For low-density leaf regions, feature signals are strengthened through feature amplification via weighting (when the weight approaches 0, amplification is actually achieved by reciprocal logic, which is consistent with the negative correlation in density calculation), thereby alleviating the feature shift caused by SiO_2_-induced point cloud density heterogeneity at the data level.

##### 2.3.3.2 Module 2. Channel attention normalization layer for SA layer enhancement in cucumber drought phenotyping

Cucumber exhibits distinct global morphological characteristics under drought stress, such as large-scale leaf curling, uneven chlorophyll distribution, and altered stem lignification. These traits often require coordinated representation of global statistical information and local details. To accurately capture morphological and physiological differences in cucumber seedling point clouds under drought stress, this study addresses a critical limitation of the PointNet++-SSG backbone: its exclusive reliance on max pooling during feature aggregation, which easily overlooks global structural information including leaf curling and stem wilting. We propose the Channel Attention Normalization Layer (CANL) to improve the feature aggregation process of the Set Abstraction (SA) module with attention enhancement (referring to Zhang Chi et al. (2024) — Improved PointNet++ Based on Feature Deviation and Attention Mechanism) [19]. A dynamic channel attention–global average pooling–residual fusion enhancement module is embedded in the single-scale feature aggregation stage. The complete implementation steps of the CANL module are detailed in Equations 9–13.

Let the point cloud feature output by the SA module after single-scale Ball Query neighborhood search and PointNet feature extraction be: $F\in {\mathbb{R}}^{B\times C\times N}$ where *B* is the batch size, *C* is the dynamically changing number of feature channels (automatically generated by network levels, not fixed), and *N* is the number of point clouds in the single-scale neighborhood. The three-stage pipeline of the improved module is as follows:

(1) Dynamic global average pooling (capturing global morphological features of drought-stressed cucumbers).

To compensate for the limitation of max pooling that only focuses on local extrema, global average pooling is first applied to local feature *F* along the point dimension, aiming to extract global statistical features representing the overall drought status of cucumber seedlings, such as plant compactness and average leaf curling degree.

(9) $$F_{gap}=\frac{1}{N}\overset{N}{\underset{i=1}{\sum }}F\;(:,:,i)\;F_{gap}\in {\mathbb{R}}^{B\times C\times 1}$$

The global feature value of each channel corresponds to the average response of all points under that channel. This operation dynamically adapts to an arbitrary number of channels C without manual dimension setting, and can stably extract global drought features of cucumber seedling point clouds at different levels. It effectively reflects global drought phenotypes such as the overall wilting trend and canopy shrinkage amplitude of cucumber seedlings, providing a global reference for subsequent attention weighting.

(2) Dynamic channel attention generation (adaptively enhancing drought-sensitive channels).

A lightweight squeeze-and-excitation attention network is constructed according to the dynamically obtained number of channels *C* to adaptively weight feature channels related to cucumber drought response. First, the global feature *F_gap_* is fed into a dynamic network composed of two 1×1 convolutions, whose dimensions automatically match the number of channels *C*.:

(10) $$F_{conv1}=\delta \left(Conv_{1\times 1}\left(F_{gap},C_{mid}=\left[C/4\right]\right)\right)$$

(11) $$W=\sigma \left(Conv_{1\times 1}\left(F_{conv1},C_{out}=C\right)\right)$$

where, δ denotes the ReLU nonlinear activation function, σ denotes the Sigmoid activation function, and $W\in {\mathbb{R}}^{B\times C\times 1}$ is the dynamically generated channel attention weight. The weight values adaptively enhance leaf and stem feature channels highly correlated with cucumber drought phenotypes, suppress background noise and redundant channels, and realize importance recalibration in the channel dimension.

(3) Residual feature fusion (preserving original features and enhancing drought signals).

To avoid distortion of original geometric features caused by attention weighting and ensure the stability of feature propagation, a residual weighting strategy is adopted to fuse the attention weights with the original features, formulated as:

(12) $$F_{att}=F⊗W+F$$

where ⊗ represents element-wise multiplication under broadcasting. This structure maintains dimension alignment under dynamic channel conditions, preserving the original geometric features of cucumber seedlings while significantly enhancing the representation of key phenotypic traits such as leaf curling and stem wilting under drought stress.

(4) Layer normalization for feature calibration (unifying distribution and alleviating training instability).

To address numerical shift and gradient instability caused by the stacking of density enhancement and attention modules, a layer normalization (LayerNorm) strategy is inserted between the two modules to unify the feature distribution range:

(13) $$F_{enhanced}=\gamma \cdot \frac{F_{att}-\mu }{\sqrt{\sigma^{2}+\epsilon }}+\beta$$

Where *μ* and σ^2^ are the mean and variance of *F_att_* over the channel and point dimensions, respectively, $𝝐=10^{-5}$ is a small constant to avoid division by zero, and *γ* and *β* are learnable scale and shift parameters. This strategy eliminates feature distribution conflicts caused by multi-module stacking, realizes deep coupling and collaborative enhancement of local density features and global semantic features, and improves training stability and segmentation accuracy of the model in small-sample cucumber drought point cloud tasks.

##### 2.3.3.3 Module 3. Drought-aware hybrid loss function for leaf-stem binary classification with imbalanced data

Cucumber seedling point clouds, under the dual effects of SiO_2_ modification and drought stress, suffer from severe class imbalance: extremely sparse points in the stem region and an excessively high proportion of points in the leaf region. Traditional cross-entropy (CE) loss supervises all samples with equal weights, causing the model to tend to learn the more prevalent leaf features during training, which severely reduces the segmentation precision and IoU of small stem targets. Although the single Dice loss optimizes small-target segmentation by calculating the overlap between predictions and ground truth, it is highly sensitive to noise in small-sample data and easily leads to gradient explosion and convergence oscillation, failing to ensure stable model training. In addition, leaf-stem segmentation of cucumber seedling point clouds is a fine-grained task in agricultural scenarios, where stem segmentation accuracy directly affects the quantitative analysis of drought stress levels. Therefore, this study proposes a Drought-Aware Hybrid Loss function (DHL) for plant point clouds, which fuses weighted cross-entropy and Dice loss. Through triple mechanisms of class weight redistribution, small-target segmentation optimization, and dynamic loss fusion, it solves the problem of insufficient supervision of small stem targets in cucumber seedling leaf-stem segmentation and improves the practicality and accuracy of the model in agricultural plant point cloud segmentation tasks. The complete implementation steps of the DHL module are detailed in Equations 14–18.

Let the predicted probability tensor output by the model in the cucumber seedling point cloud segmentation task be $P\in {\mathbb{R}}^{B\times K\times N}$, Let the predicted probability tensor output by the model in the cucumber seedling point cloud segmentation task be $Y\in {\mathbb{R}}^{B\times K\times N}$, where *B* denotes the batch size, *K* = 2 is the number of segmentation classes (*k* = 0 for leaf class, *k* = 1 for stem class), and *N* is the number of point clouds per cucumber seedling. A small constant $𝝐=10^{-8}$ is introduced to avoid invalid logarithmic operations or division by zero.

(1) Weighted cross-entropy (WCE) loss.

To compensate for the lack of supervision weight caused by insufficient stem samples, the inverse class frequency method is used to assign differentiated weights to different classes and construct the weighted cross-entropy loss. First, the number of point clouds of each class in the dataset is counted, and the class weighting coefficients are calculated:

(14) $$w_{k}=\frac{N_{total}-N_{k}}{N_{total}}$$

Where *N_total_* is the total number of point clouds in the dataset, and *N_k_* is the number of point clouds of the *k*-th class. For the stem class (*k* = 1), since *N*_1_ is much smaller than *N_total_*, the weighting coefficient *w*_1_ will be significantly larger than that of the leaf class *w*_0_, thereby increasing the supervision weight of stem samples in loss calculation.

Based on the class weighting coefficients, the weighted cross-entropy loss is formulated as:

(15) $$WCE=-\frac{1}{N}\overset{B}{\underset{b=1}{\sum }}\overset{N}{\underset{n=1}{\sum }}\overset{1}{\underset{k=0}{\sum }}w_{k}\cdot Y_{b,k,n}\cdot \log(P_{b,k,n}+\epsilon )$$

By reassigning weights, this loss encourages the model to pay more attention to feature learning of small stem targets during training, alleviating model bias caused by class imbalance.

(2) Dice loss.

Considering the segmentation characteristics of small stem targets, a Dice loss for the stem class is designed to directly optimize the overlap between the predicted region and the ground-truth label, formulated as:

(16) $$Dice=1-\frac{2\sum_{b=1}^{B}\sum_{n=1}^{N}Y_{b,1,n}\cdot P_{b,1,n}+\epsilon }{\sum_{b=1}^{B}\sum_{n=1}^{N}Y_{b,1,n}+\sum_{b=1}^{B}\sum_{n=1}^{N}P_{b,1,n}+\epsilon }$$

This formula calculates the Dice coefficient only for the stem class (*k* = 1), preventing the high proportion of the leaf class from masking segmentation errors of the stem. It can precisely improve the recall rate and IoU of the stem region and solve the core problem of missed detection in small-target segmentation.

(3) Design of adaptive fusion loss function.

Although the single weighted cross-entropy loss can alleviate class imbalance, it still relies on logarithmic supervision of probability distribution and provides insufficient optimization for small-target boundary segmentation. On the other hand, although single Dice loss is favorable for small targets, it is susceptible to noise and leads to unstable training. Therefore, an IoU-aware adaptive balance factor *α* ∈ [0, 1] is introduced to dynamically fuse weighted cross-entropy and Dice loss. The total loss function is formulated as:

(17) $${ℒ}_{total}=\alpha \cdot WCE+\left(1-\alpha \right)\cdot Dice$$

The balance factor *α* is updated in real time according to the IoU values of stems and leaves during training, and its calculation formula is:

(18) $$\alpha =\frac{IoU_{stem}}{IoU_{stem}+IoU_{leaf}}$$

Where *IoU_stem_* is the IoU of the stem class and *IoU_leaf_* is the IoU of the leaf class. When the stem IoU is low, *α* decreases, increasing the proportion of Dice loss to strengthen the segmentation optimization of small stem targets. When the leaf IoU is low, *α* increases, raising the proportion of weighted cross-entropy loss to ensure classification accuracy of the leaf region, achieving complementary advantages of the two losses.

### 2.4 Model training parameters and evaluation metrics

#### 2.4.1 Model training parameters

All model training and validation experiments in this study were built on a high-performance computing platform. The specific configuration of the experimental environment is shown in **Table 1**, and the model training parameters are listed in **Table 2**.

**Table 1:** Hardware and software environment configuration table for DDCANet model training.

Category	Configuration item	Specific parameters
Hardware Environment	Operating system	Windows 11 (64-bit, 23H2)
	Processor	13th Gen Intel Core i7-13700H (2.40 GHz)
	RAM	16.0 GB DDR4
	Graphics card	NVIDIA GeForce RTX 4060 Laptop GPU(8 GB GDDR6)
Software Environment	Integrated Development environment (IDE)	PyCharm Professional
	Programming language	Python 3.8
	Deep learning framework	PyTorch 1.13.1, torchvision 0.14.1, torchaudio 0.13.1
	Parallel computing & acceleration library	CUDA 11.6, cuDNN 8.4

**Table 2:** Core training hyperparameter settings of the DDCANet model.

Training parameters	Specific value
Batch Size	16
Epoch	100
Optimizer	Adam
Learning Rate	0.001
Decay Rate	10^-4^
Npoint	4096

Meanwhile, to prevent overfitting during model training and improve training efficiency, an early stopping strategy based on validation loss is adopted in this experiment: when the decrease in validation loss is less than 1×10^-5^ for 10 consecutive training epochs, the model is judged to have converged and training is automatically terminated. Meanwhile, the optimal model weights with the lowest loss during training are preserved to ensure model stability and generalization ability.

#### 2.4.2 Evaluation metrics

To quantitatively evaluate the performance of the improved PointNet++ model in the segmentation task of cucumber drought phenotypic point clouds, this paper selects leaf IoU, stem IoU, mean Intersection over Union (mIoU), and overall point-wise Accuracy as core evaluation metrics (Sarker et al., 2024). Analysis is carried out from three dimensions: single-class segmentation accuracy, multi-class comprehensive segmentation performance, and point-level overall classification correctness. The mathematical definitions of each metric are detailed in Equations 19–21, as follows:

Single-class Intersection over Union (IoU) is a key metric for evaluating single-class segmentation accuracy in point cloud semantic segmentation, characterizing the overlap between the predicted region and the ground-truth region of a certain class of point clouds. The IoU calculation formulas for the leaf and stem classes are unified as:

(19) $$IoU_{k}=\frac{TP_{k}}{TP_{k}+FP_{k}+FN_{k}}$$

where *TP_k_* is the number of correctly predicted point clouds for the *k*-th class, *FP_k_* is the number of non-*k* point clouds incorrectly predicted as class *k*, and *FN_k_* is the number of *k*-th class point clouds incorrectly predicted as other classes, where *k* denotes *leaf* or *stem*.

Mean Intersection over Union (mIoU) is the arithmetic mean of IoU values across all classes. It comprehensively reflects the model’s overall segmentation ability for multi-class point clouds and is a core evaluation metric in point cloud semantic segmentation tasks. Its calculation formula is:

(20) $$mIoU=\frac{1}{\text{C}}\sum_{\text{k}=1}^{\text{C}}{\text{IoU}}_{\text{k}}$$

Where *C* is the total number of classes in point cloud segmentation (in this paper, *C* = 2, corresponding to the leaf and stem classes).

Overall point accuracy (Acc) measures the proportion of all point clouds correctly classified, reflecting the overall point-level classification performance of the model. The calculation formula is:

(21) $$Point\;Accuracy=\frac{\sum_{k=1}^{C}TP_{k}}{N}$$

Where *N* is the total number of cucumber point clouds to be segmented, and $\sum_{k=1}^{C}TP_{k}$ is the sum of correctly predicted point clouds across all classes.

### 2.5 Phenotypic parameter calculation

#### 2.5.1 Point cloud data preprocessing

To eliminate the interference of noise and invalid data on subsequent feature calculation, we performed invalid point removal and statistical filtering denoising:

Invalid point removal involves traversing the original point cloud data and deleting invalid points with missing values (NaN) and coordinates beyond the reasonable range, ensuring that the point cloud data only contains the target plant region. The calculation formula is detailed in Equation 22, as follows:

(22) $$P_{valid}=\left\{\left(x,y,z\right)|x\in \left[-0.5,0.5\right],\;y\in \left[-0.5,0.5\right],\;z\in \left[0,1.0,\right]x,y,z\neq NaN\right\}$$

where *P_valid_* is the set of valid point clouds, and (*x*, *y*, *z*) is the 3D coordinate of a single point cloud.

Then, a statistical filtering algorithm is used to remove isolated noise points. The number of neighborhood points *k* = 10 and the standard deviation multiplier is set to 1.0. Outliers are selected by calculating the average neighborhood distance of each point, with the formulas detailed in Equations 23, 24, as follows:

(23) $$d_{i}=\frac{1}{k}\overset{k}{\underset{j=1}{\sum }}\sqrt{{\left(x_{i}-x_{j}\right)}^{2}+{\left(y_{i}-y_{j}\right)}^{2}+{\left(z_{i}-z_{j}\right)}^{2}}$$

(24) $$P_{filter}=\left\{\left(x_{i},y_{i},z_{i}\right)|\left|d_{i}-\bar{d}\right|\leq 1.0\times \sigma_{d}\right\}$$

where *d_i_* is the average neighborhood distance of the *i*-th point, $\bar{d}$ is the mean of the average neighborhood distances of all points, σ*_d_* is the standard deviation of the average neighborhood distances, and *P_filter_* is the purified point cloud set after filtering.

#### 2.5.2 Instance segmentation

Based on the filtered point cloud data, we adopt the KDTree-accelerated Euclidean clustering algorithm to achieve instance segmentation of individual cucumber plants.First, spatial indexing is constructed by inputting *P_filter_* into KDTree to build a 3D spatial index, improving the efficiency of neighborhood point search. Then, clustering parameters are set: neighborhood search radius *ϵ* = 0.12 m, minimum clustering points min_*pts* = 5 (to filter noise clusters).Finally, breadth-first clustering is performed. For each unvisited point *P_i_* the neighborhood point set *N_ϵ_*(*p_i_*) within its *ϵ* range is queried. If $\left|N_{\epsilon }\left(p_{i}\right)\right|\geq \text{min}\_pts$, the points are marked as the same cluster *C_m_* (where *m* is the cluster index).

#### 2.5.3 Calculation of core phenotypic traits

Based on the point cloud set of a single plant, four core phenotypic traits are calculated, with detailed methods and formulas shown in **Table 3**.

**Table 3:** Calculation methods and formulas of core 3D phenotypic traits of cucumber seedlings.

Feature	Calculation method and formula
Plant height (*H*)	Longitudinal growth height, defined as the difference between the maximum and minimum Z-coordinates: $\text{H}=\max({\text{z}}_{\text{i}})-\min({\text{z}}_{\text{i}}),\text{ i}=1,2,\ldots ,\text{n}$
Volume (*V*)	The 3D convex hull volume is calculated using the convex hull algorithm: $\text{V}=\frac{1}{6}\sum_{\text{k}=1}^{\text{m}}\left|\left({\text{x}}_{\text{k}2}-{\text{x}}_{\text{k}1})\cdot [({\text{y}}_{\text{k}3}-{\text{y}}_{\text{k}1})\times ({\text{z}}_{\text{k}3}-{\text{z}}_{\text{k}1})\right]\right|$m: Number of triangular faces in the convex hull ·: Dot product ×: Cross product
Compactness (C)	Morphological compactness (normalized): $\text{C}=\frac{{\text{V}}_{\text{plant}}}{{\text{V}}_{\text{hull}}}\times 100\%$V_plant_: Actual volume calculated by voxelization method (voxel resolution = 0.001 m) V_hull_: Convex hull volume
Crown width (W_X_/W_Y_)	Transverse growth range: ${\text{W}}_{\text{X}}=\max({\text{x}}_{\text{i}})-\min({\text{x}}_{\text{i}}),\;{\text{W}}_{\text{Y}}=\max({\text{y}}_{\text{i}})-\min({\text{y}}_{\text{i}})$

#### 2.5.4 Statistical analysis of phenotypic data

All phenotypic data in this study were obtained from three biological replicates, and the results are presented as Mean ± Standard Error (Mean ± SE). The error bars in the statistical figures represent the standard error (SE), while the 95% Confidence Interval (95% CI) is adopted in the regression analysis figures. This study is an exploratory drought phenotyping research based on 3D point cloud technology, focusing on analyzing the dynamic variation trends of phenotypic indicators under different SiO_2_ concentrations and treatment durations. Descriptive statistics and trend comparison methods were used to reveal the difference patterns among treatments, thereby supporting the determination of the optimal concentration. Subsequent studies will employ a larger sample size and combine two-way repeated-measures analysis of variance (two-way repeated-measures ANOVA) with multiple comparisons to conduct more systematic statistical tests.

### 2.6 Ablation experiments

**Figure 4:**
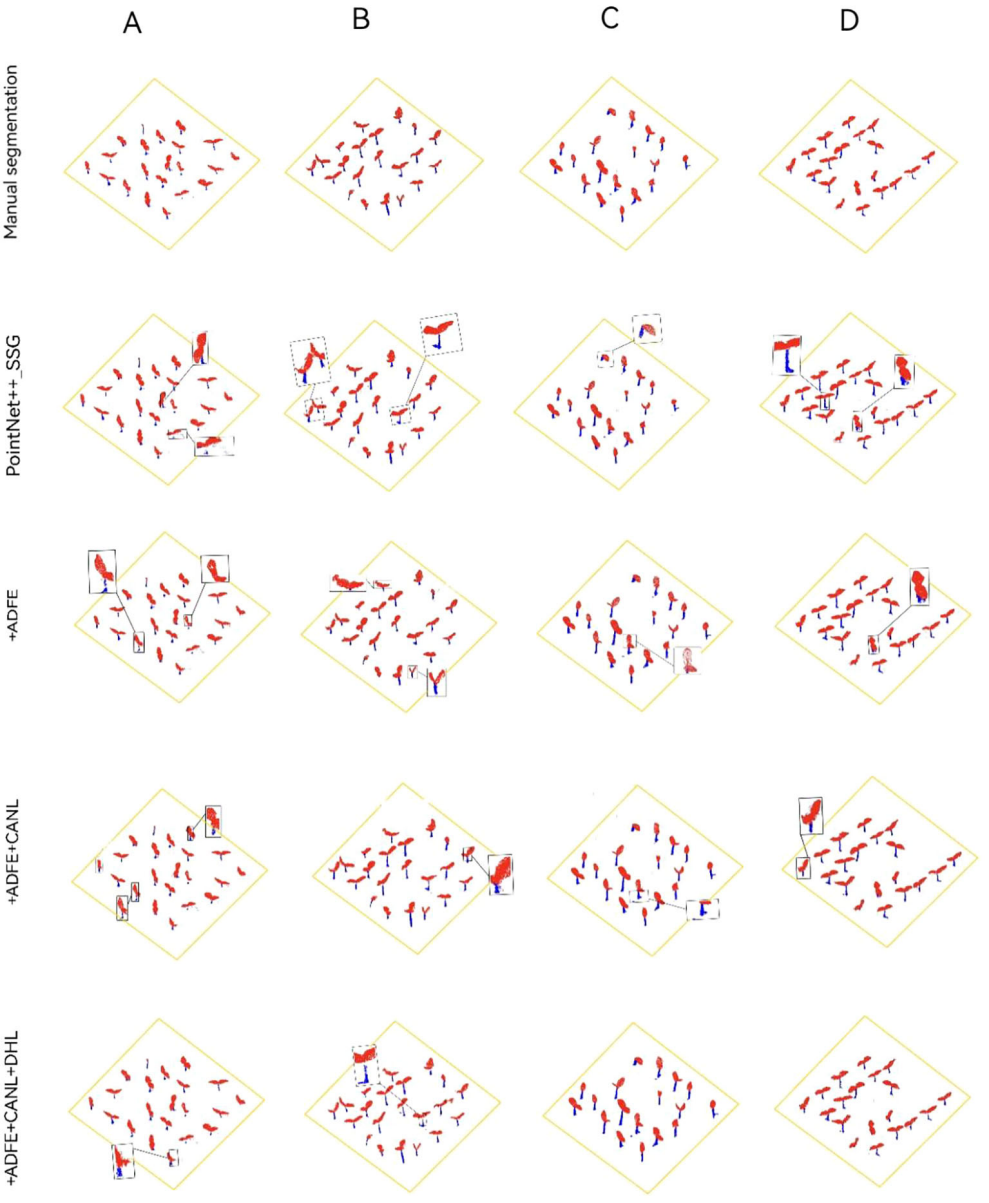
Visual comparison of semantic segmentation results of cucumber seedling point clouds in ablation experiments [samples **(A–D)**].

As shown in **Table 4** and **Figure 4**, an incremental ablation study is designed in this study. Using PointNet++_SSG as the baseline model, three core improved modules (ADFE, CANL, DHL) are incorporated sequentially to verify the individual contribution and combined synergy of each component. All experiments were evaluated via 3-fold cross-validation. Results are reported as the mean ± standard deviation over the three folds, ensuring the consistency and robustness of our findings. Leaf IoU, Stem IoU, mIoU, and Acc are selected as core evaluation metrics, with a focus on performance improvements for small targets such as stems. Meanwhile, consistent experimental settings (dataset, training hyperparameters, hardware configuration) are maintained to eliminate interference from irrelevant variables and accurately quantify the improvement value of each module.

**Table 4:** Performance evaluation indicators of each module in ablation experiments of the DDCANet model.

Metrics model	Leaf IoU(%)	Stem IoU(%)	mIoU(%)	Acc(%)
PointNet++-SSG	90.8 ± 0.28	74.0 ± 0.41	82.46 ± 0.35	92.72 ± 0.29
+ADFE	92.2 ± 0.25	76.7 ± 0.38	84.47 ± 0.32	93.81 ± 0.26
+ADFE+CANL	93.6 ± 0.23	79.7 ± 0.36	86.62 ± 0.31	94.86 ± 0.26
+ADFE+CANL+DHL	94.4 ± 0.21	83.6 ± 0.45	89.01 ± 0.32	95.63 ± 0.25

After adding ADFE to the baseline model, Leaf IoU increases from 90.8% to 92.2%, Stem IoU from 74.0% to 76.7%, and mIoU and Acc also rise synchronously. This indicates that the module can specifically enhance feature representation in low-density stem regions within SiO_2_-induced heterogeneous density point clouds, alleviating the suppression of small-target features caused by the sensitivity of traditional PointNet++ to local density variations. From the data perspective, it improves the model’s robustness in representing heterogeneous point clouds, thus achieving basic improvements in both leaf and stem segmentation accuracy.

With the addition of CANL, the Stem IoU increases by 3.0% (from 76.7% to 79.7%), which is a larger gain than that of leaf segmentation (from 92.2% to 93.6%). This demonstrates that the module resolves feature distribution conflicts and gradient instability caused by multi-module stacking, while achieving deep coupling between local density features and global semantic features, with a more prominent optimization effect on fine-structured small targets such as stems.

After incorporating DHL, Stem IoU is further enhanced by 3.9% (79.7% → 83.6%), and mIoU exceeds 89%. This shows that DHL can increase the supervision weight of stem samples through a class-weighting strategy, optimize the overlap between predicted regions and ground-truth labels by combining the Dice coefficient, and adaptively balance the convergence speed of cross-entropy and the segmentation accuracy of Dice. Consequently, it effectively alleviates model bias caused by sample imbalance and promotes a substantial leap in stem segmentation performance.

The ablation experiments verify the hierarchical synergistic effects of the three improved modules: ADFE lays the foundation for heterogeneous point cloud feature extraction at the data representation level (Zhang et al., 2025), CANL enhances the effectiveness and stability of feature fusion at the network structure level, and DHL addresses supervision deficiencies caused by class imbalance at the loss function level (Niu et al., 2024). Together, they form a full-chain optimization system of “data–structure–supervision”. Experimental results show that the progressive integration of each module yielded consistent performance gains. Specifically, the final combined model outperformed the baseline by 9.6% in Stem IoU, 6.55% in mIoU, and 2.91% in overall accuracy (Acc). All standard deviations from the 3-fold cross-validation were below 0.5%, confirming the stability and reliability of the results. These findings demonstrate that each module was designed to directly address key challenges in cucumber drought phenotyping point cloud segmentation—namely density heterogeneity, feature conflicts, and class imbalance—thereby validating the effectiveness and rationality of our proposed improvements.

### 2.7 Comparison of semantic segmentation results of cucumber seedlings using different models

To verify the superior segmentation performance of the improved DDCANet model, this study selects three classic point cloud segmentation models—PointNet, PointNet++-MSG, and PointNet++-SSG—as baselines for comparison, with analyses conducted from both quantitative metrics and qualitative visualization.

These three classical models in point cloud semantic segmentation are chosen as comparative methods, representing typical design paradigms: no local feature extraction (PointNet), multi-scale local feature fusion (PointNet++-MSG), and single-scale local feature aggregation (PointNet++-SSG). A progressive comparison is performed against the proposed DDCANet. The experiments adopt Leaf IoU, Stem IoU, mIoU, and Acc as quantitative evaluation indicators. Meanwhile, visualization results are used to analyze the segmentation accuracy of the model for small stem targets and leaf regions, as well as its ability to suppress background noise.

#### 2.7.1 Quantitative comparison

**Table 5** summarizes the quantitative segmentation performance of all models, which were evaluated via 3-fold cross-validation with results presented as mean ± standard deviation. On the cucumber seedling point cloud dataset established in this study, DDCANet outperformed mainstream baselines including PointNet++ across all four key metrics. Notably, it achieved consistent gains in mean mIoU and demonstrated sustained performance advantages as well as greater robustness in the challenging stem-leaf class-imbalanced segmentation task.

**Table 5:** Evaluation Indicators of segmentation performance for different models.

Metrics model	Leaf IoU(%)	Stem IoU(%)	mIoU(%)	Acc(%)
PointNet	77.8 ± 0.35	53.1 ± 0.52	65.45 ± 0.42	82.24 ± 0.36
PointNet++-MSG	82.3 ± 0.32	59.7 ± 0.48	71.03 ± 0.38	86.01 ± 0.33
PointNet++-SSG	90.8 ± 0.28	74.0 ± 0.41	82.46 ± 0.35	92.72 ± 0.29
DDCANet	94.4 ± 0.21	83.6 ± 0.45	89.01 ± 0.32	95.63 ± 0.25

Compared with PointNet, DDCANet achieves improvements of 16.6% in Leaf IoU, 30.5% in Stem IoU, 23.56% in mIoU, and 13.39% in Acc. PointNet relies solely on global max pooling for feature extraction and completely ignores local neighborhood information. It fails to capture the structural differences between cucumber seedling leaves and stems, and its representation ability for small stem targets is severely insufficient, resulting in the lowest performance among all models.

Compared with PointNet++-MSG, the latter extracts local features via multi-scale grouping, yielding a 5.58% mIoU improvement over PointNet. However, without optimization for the density heterogeneity of cucumber seedling point clouds, multi-scale feature fusion introduces redundant information, leading to lower performance than the single-scale PointNet++-SSG. In contrast, DDCANet increases Stem IoU by 23.9% relative to PointNet++-MSG, demonstrating its strong adaptability to heterogeneous density point clouds.

Compared with the baseline model PointNet++-SSG, DDCANet improves Leaf IoU by 3.6%, Stem IoU by 9.6%, and mIoU by 6.55%. Although PointNet++-SSG resolves basic local feature extraction, it is sensitive to SiO_2_-induced density heterogeneity, suffers from global information loss, and does not address class imbalance, all of which form performance bottlenecks. DDCANet achieves substantial performance gains through targeted optimizations in three dimensions: data representation, network architecture, and loss supervision.

#### 2.7.2 Qualitative comparison

**Figure 5:**
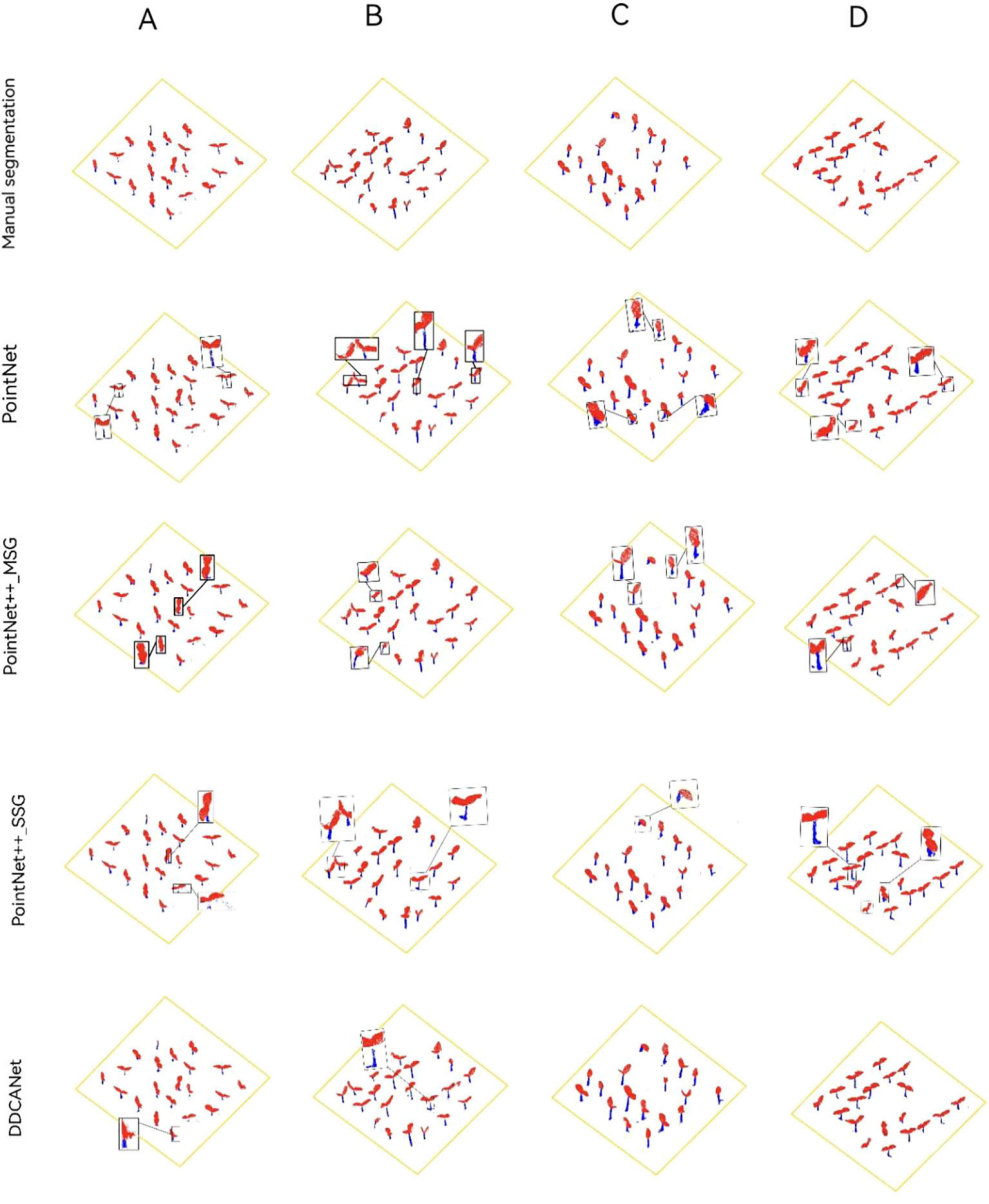
Visual comparison of semantic segmentation results of cucumber seedling point clouds using different models [samples **A–D)**].

**Figure 5** shows the visual comparison between manual annotations and segmentation results of various models for four representative cucumber seedling samples (A–D). Key defective regions are marked with boxes, which intuitively reflect the segmentation differences among models.

The segmentation results of PointNet exhibit severe missing regions in the stem area and serious fragmentation at leaf edges, with frequent misclassification of background points as foreground. The deviation from manual annotations is the largest, confirming its inherent drawback of insufficient local feature extraction capability.

In PointNet++-MSG, the problem of stem missing is alleviated, but over-segmentation occurs in dense leaf areas, and the boundaries between stems and leaves are blurred and adhered, indicating the interference of redundant multi-scale feature fusion on segmentation accuracy.

PointNet++-SSG preserves the overall contour of the stem, but breaks appear in low-density stem regions, and leaf details are coarsely segmented, reflecting its sensitivity to heterogeneous density point clouds and loss of global semantic information.

The segmentation results of DDCANet are almost completely consistent with manual annotations. Small target stems are completely retained, leaf regions are segmented continuously and completely, and background noise is accurately filtered out. The restoration of key details (such as fine stem branches and adhered leaf boundaries) is far superior to that of the comparative models, which fully verifies the effectiveness of the improved modules. It should be noted that the model still exhibits a few typical errors under certain complex scenarios. For example, when leaves are severely occluded, stems are extremely thin, or there is significant noise, the model may suffer from blurred local boundaries or minor under-segmentation errors. This indicates that there is still room to improve the model’s robustness to extreme scales and occluded scenes.

The combined analysis of quantitative indicators and qualitative visualization shows that classical point cloud segmentation models suffer from drawbacks such as insufficient local feature extraction, redundant feature fusion, and sensitivity to heterogeneous density and small targets, making them unsuitable for the complex segmentation scenarios of cucumber seedling point clouds (Zarei et al., 2024). In contrast, the DDCANet proposed in this study integrates three key modules: adaptive density-aware feature enhancement, channel attention and feature normalization co-optimization, and weighted cross-entropy–Dice fusion loss. These improvements address the above issues across the entire pipeline from data representation and network architecture to loss supervision, ultimately achieving high-quality segmentation with no missed segmentation, no over-segmentation, and precise boundary localization.

## 3 Results

### 3.1 Experimental design

This study investigated the effects of priming with different concentrations of silicon dioxide (SiO_2_) nanoparticle solution on drought tolerance in cucumber seedlings. A 60 g/L PEG solution was used to simulate drought conditions (Liu et al., 2024), and six drought concentration gradients of SiO_2_ nanoparticles (0, 20, 40, 60, 80, 100 mg/L) were set, among which the deionized water group with a concentration of 0 served as the blank control group (CK). No non-drought control group with sole SiO_2_ application (including 0 mg/L) under normal water conditions was established, and all intergroup comparisons were performed under the background of drought stress. The dose–effect relationship between SiO_2_ nanoparticle concentration and cucumber drought tolerance was clarified through gradient concentration treatments.

**Figure 6:**
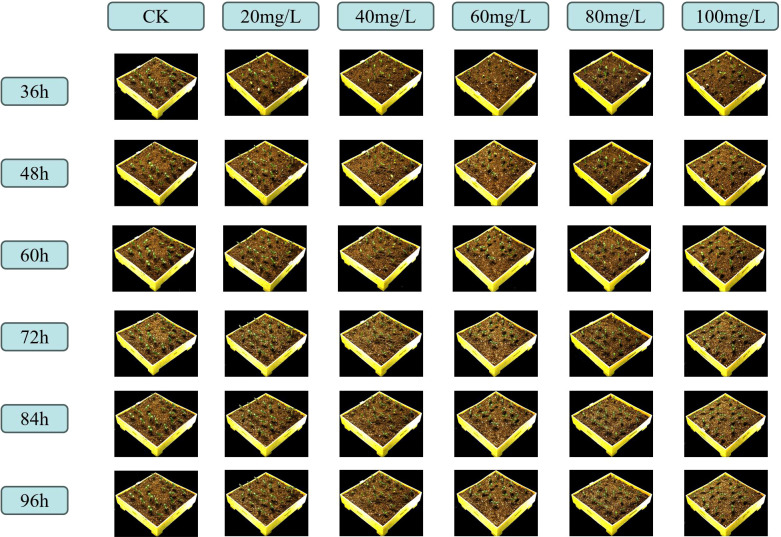
Axial photographic results of cucumber seedlings during growth (36h–96h).

**Figure 7:**
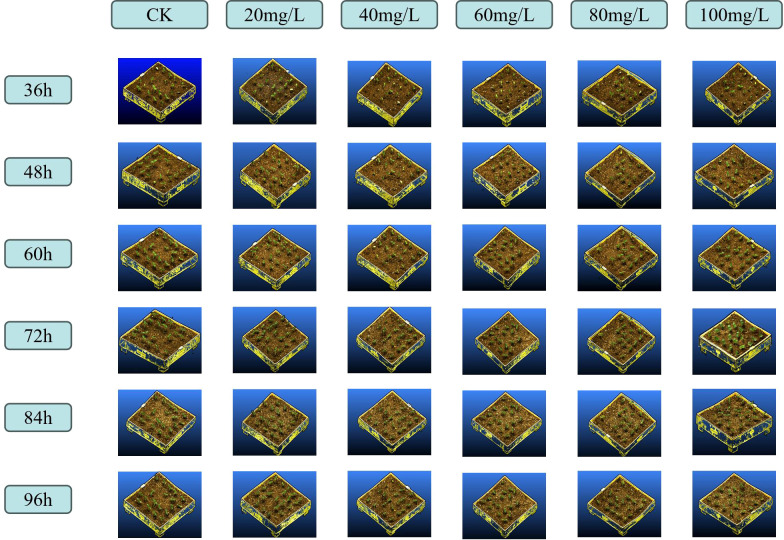
3D point cloud reconstruction results of cucumber seedlings during growth (36h–96h).

During the experiment, 25 cucumber seeds were selected for each concentration gradient, and three biological replicates were set to reduce experimental errors and ensure data reliability. In the data collection stage, no seedling emergence was observed within 24 hours after sowing (Liu et al., 2023). The first phenotypic data collection was set at 36 hours after sowing (Ren and Wang, 2024), and subsequent image collection and point cloud reconstruction were performed every 12 hours (**Figure 6** shows axial photographic images of the cucumber seedling growth process, and **Figure 7** shows 3D point cloud reconstruction images of the growth process) until the experiment ended at 96 hours after sowing.

**Figure 8:**
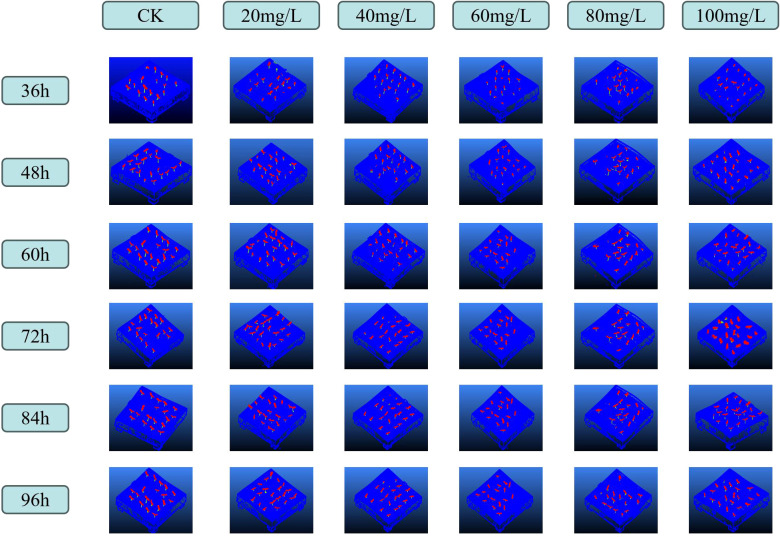
Semantic segmentation results of 3D point cloud of cucumber seedlings based on the DDCANet model (36h–96h).

**Figure 9:**
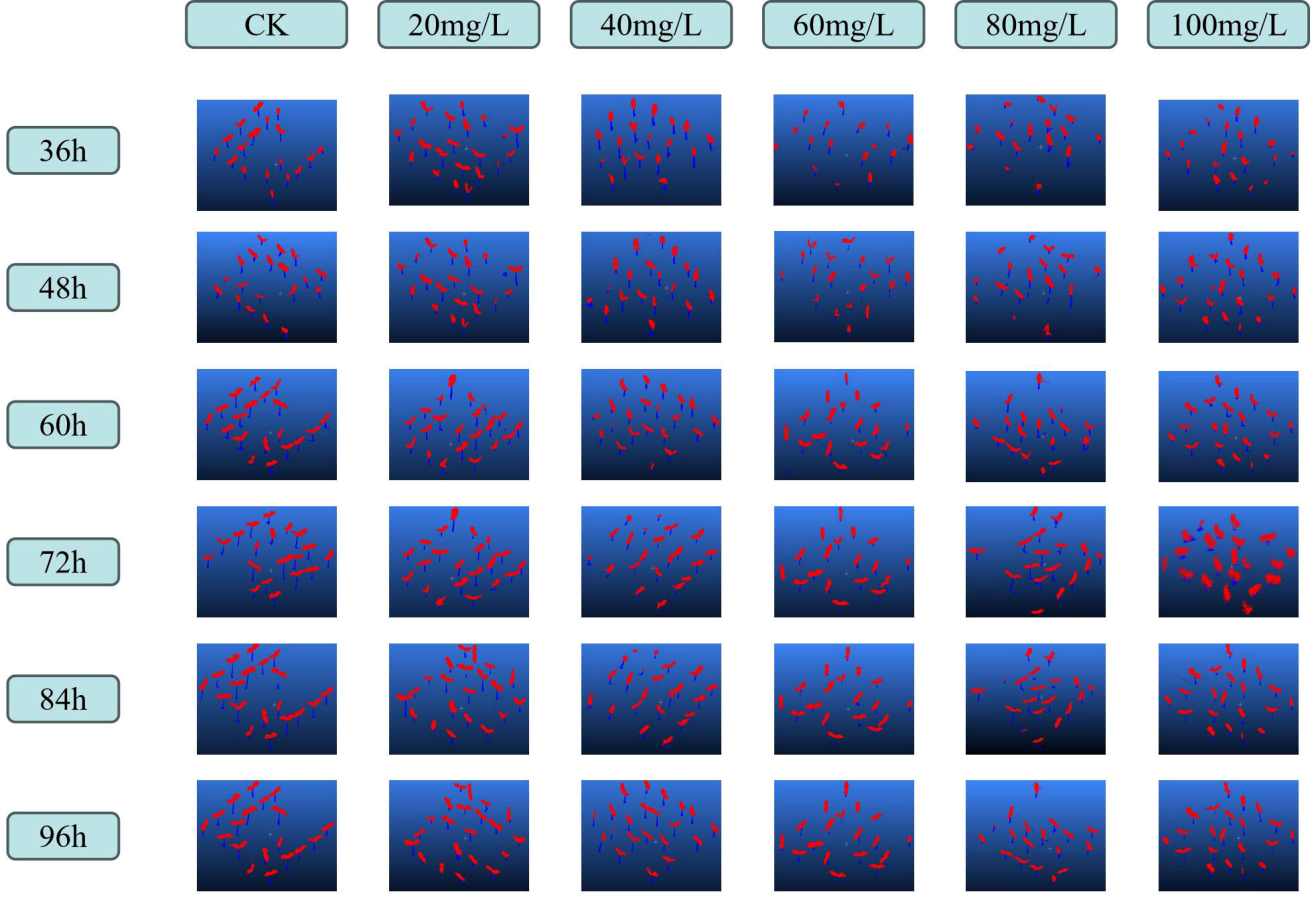
Semantic segmentation results of 3D point cloud of cucumber seedlings after pot removal (36h–96h).

During feature extraction, semantic segmentation of cucumber plants was completed based on the improved DDCANet model (**Figure 8** shows 3D point cloud data images of cucumber seedlings segmented by the DDCANet model (Xing, 2024; Yang et al., 2023), and **Figure 9** shows semantic segmentation images of cucumber seedlings after pot removal to improve algorithm efficiency). Then, instance segmentation of plants was realized using the Euclidean clustering algorithm, and key phenotypic indicators such as plant height, volume, compactness, and crown width (X and Y directions) were accurately extracted through phenotypic trait algorithms. Quantitative analysis of phenotypic differences in cucumber seedlings under different SiO_2_ nanoparticle concentrations revealed the phenotypic response rules of SiO_2_ nanoparticles regulating drought tolerance in cucumbers.

### 3.2 Effects of different SiO_2_ nanoparticle priming concentrations on phenotypic traits of cucumber seedlings

**Figure 10:**
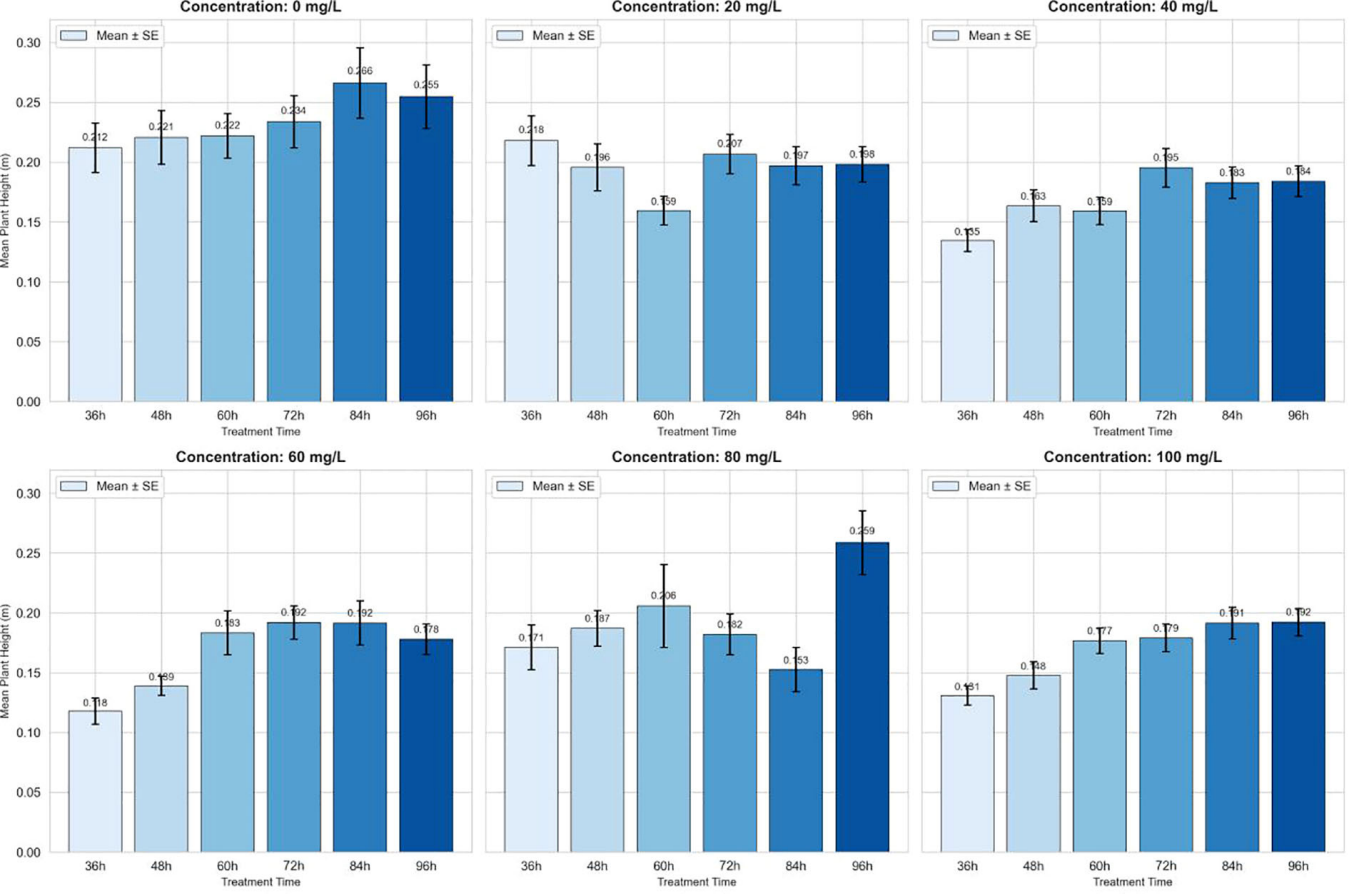
Dynamic change trend of plant height of cucumber seedlings primed with different concentrations of SiO_2_ nanoparticles (36h–96h).

As shown in **Figure 10**, under drought stress, the plant height of cucumber seedlings in the CK control group (SiO_2_ concentration 0) increased slowly and continuously with treatment time. Overall growth was significantly inhibited by drought and remained at a relatively low basic level. The 20 mg/L SiO_2_ low-concentration treatment group showed unstable height growth, with a decline in the middle stage; the overall plant height was lower than that of the blank control, indicating a weak effect in alleviating drought and promoting growth. The plant height of the 40 mg/L SiO_2_ treatment group increased steadily over time with uniform growth, and the height at each stage was close to the control group, which could slightly maintain longitudinal growth. When the concentration increased to 60 mg/L, the initial seedling height was relatively low, the growth trend was similar to the control, but the overall vigor was weaker. The 80 mg/L SiO_2_ treatment group exhibited a rapid and significant increase in plant height in the later stress stage, becoming the best-performing group among all treatments. The 100 mg/L high-concentration group showed a uniform increase in plant height, with overall vigor between those of the 40 mg/L and 80 mg/L groups. In summary, SiO_2_ regulation of cucumber seedling height under drought stress shows obvious concentration effects and time accumulation effects: low concentrations provide insufficient improvement or even inhibition, while a moderately high concentration can significantly promote late-stage height elongation. This dose-dependent response pattern is highly consistent with the findings of Zhuang et al. on the dose effect of SiO nanoparticles in drought resistance of cucumber (Zhuang et al., 2025).

Plant height depends on stem cell division, longitudinal elongation, and water balance under drought (Guo and Zhang, 2024). Drought disrupts cell turgor, inhibits internode cell elongation, and hinders height growth (Shao et al., 2018). This core inhibitory mechanism of drought on plant longitudinal growth has been systematically elaborated in the specialized study by Zhao et al. on silicon-mediated regulation of crop stem elongation (Zhao et al., 2021). Exogenous SiO_2_ can deposit on cell walls, strengthen tissue structure, reduce water transpiration loss, maintain normal cell turgor, and ensure longitudinal stem growth (Qian et al., 2022). Meanwhile, it can enhance antioxidant enzyme activity, reduce drought-induced membrane damage, and maintain normal growth-related metabolism. Meanwhile, according to the findings of Zhao et al., an appropriate concentration of SiO_2_ can significantly enhance the activity of the plant antioxidant enzyme system, alleviate drought-induced membrane lipid peroxidation damage, maintain the normal operation of growth-related metabolic pathways, and thereby provide physiological support for the sustained growth of stems. Consistent with the dose-effect rule of SiO_2_ nanoparticles on drought resistance in cucumber reported by Zhuang et al., SiO_2_ can also improve antioxidant enzyme activity, reduce drought-induced membrane damage, and promote the normal progression of growth-related metabolism (Zhuang et al., 2025). The low concentration of 20 mg/L provides insufficient silicon to continuously alleviate drought damage, and metabolic imbalance leads to mid-stage growth decline. The 40 mg/L silicon treatment only maintains basic physiological homeostasis with limited elongation-promoting effects. Based on the phenotypic results of this study and the reported optimal application concentration range of SiO_2_ for cucumber, 80 mg/L is identified as the optimal concentration suitable for cucumber seedlings under drought stress. With the prolongation of stress duration, this concentration can effectively regulate the balance of endogenous growth-related hormones such as auxin and gibberellin, accelerate internode cell elongation, and ultimately achieve a significant increase in plant height at the later stage of stress (Rakesh et al., 2024). As widely confirmed in existing studies on the application of silicon nanoparticles in crops, excessively high silicon concentrations tend to cause ion metabolic disorders and adverse physiological effects in plants (Rakesh et al., 2024; Zhuang et al., 2025). Accordingly, the growth-promoting effect of the 100 mg/L treatment was markedly inferior to that of the optimal 80 mg/L group.

**Figure 11:**
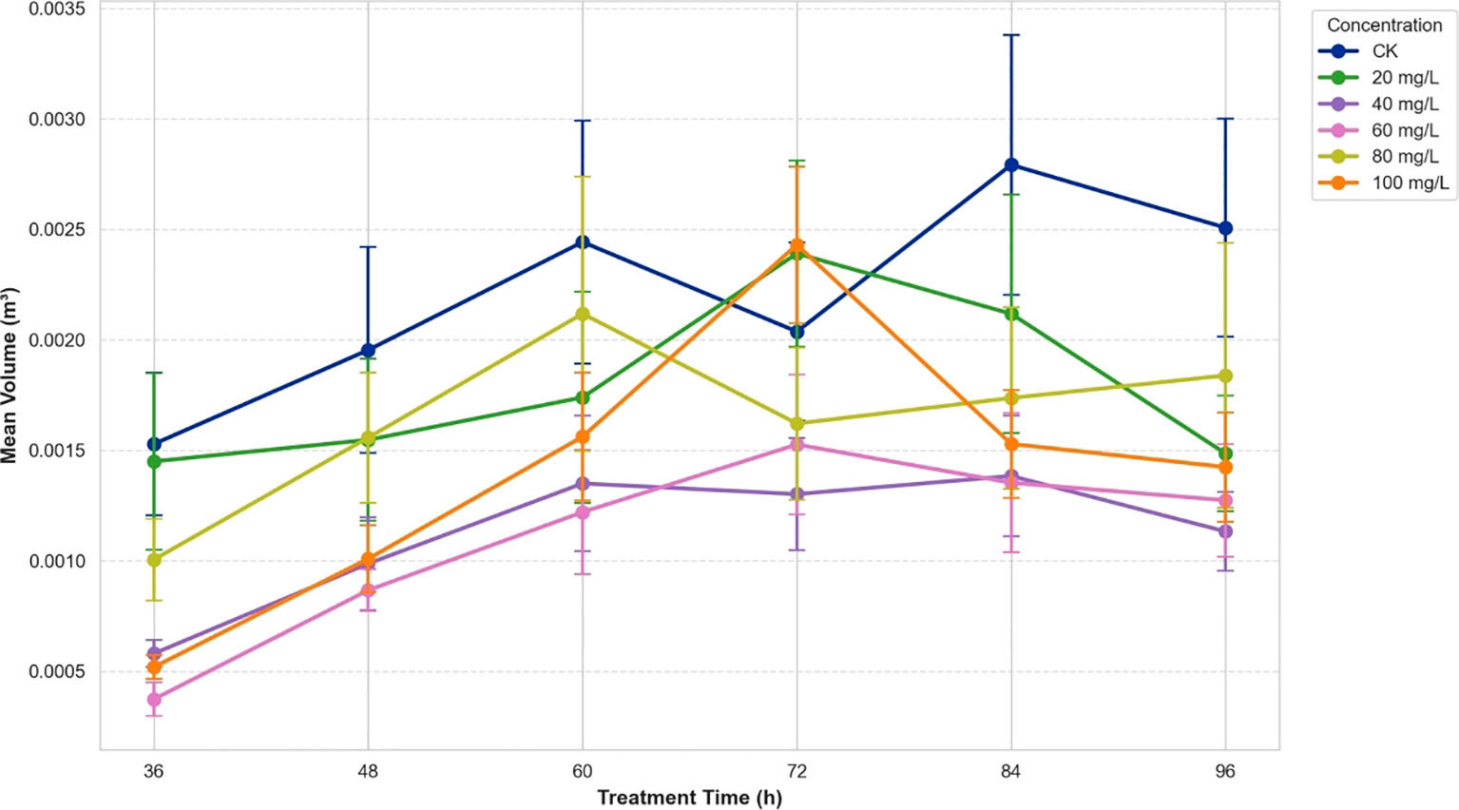
Dynamic change trend of volume of cucumber seedlings primed with different concentrations of SiO_2_ nanoparticles (36h–96h).

As shown in **Figure 11**, under drought stress, the seedling volume in the CK control group (SiO_2_ concentration 0) increased continuously with treatment time, peaked in the middle and late stages, and then declined slightly, maintaining a basic growth level overall. The seedlings treated with low-concentration SiO_2_ (20 mg/L) showed a volume change trend highly similar to that of the control group, but the volume at each time point was slightly lower than the control, with a weak effect in alleviating drought stress. The seedling volume of the 40 mg/L SiO_2_ treatment group remained at a low level overall, fluctuated and decreased in the early stage, and only recovered slowly in the later stage of stress, with the most significant growth inhibition.[13] The medium–high concentration (80 mg/L) treatment group showed a steady increase in volume over time, and the growth stability was better than that of the low- and medium-concentration groups. The seedling volume growth rate of the high-concentration (100 mg/L) SiO_2_ treatment group was significantly greater than that of other groups, and the peak value was significantly higher than all treatments, showing a strong growth-promoting effect on plant expansion. Overall, under drought stress, the regulation of SiO_2_ on cucumber seedling volume presented a nonlinear concentration effect: weak effect at low concentrations, inhibition at medium concentrations, and significant promotion at high concentrations.

According to the study by El-Mogy et al. on the regulation of cucumber plants by silicon nanoparticles under drought stress, seedling volume mainly depends on cell turgor, cell division and proliferation, and water retention capacity. Drought stress causes cell dehydration and shrinkage, decreased turgor, and inhibited tissue expansion (Zhuang et al., 2025). This inhibitory effect of drought on plant vegetative growth has been widely confirmed in studies on silicon nanoparticle-mediated drought resistance across various crops. Appropriately high-concentration SiO_2_ can enhance cellular water retention, maintain normal cell turgor, and reduce drought-induced dehydration and atrophy by increasing the content of osmotic adjustment substances such as proline and soluble sugar in plants. This osmotic regulation pathway is highly consistent with the mechanism reported by Daler et al. in their study on drought resistance of grape seedlings regulated by SiO_2_ nanoparticles (Daler et al., 2025). Meanwhile, silicon deposition on the cell wall strengthens the structure and promotes cell division and transverse growth in meristematic tissues, significantly increasing the overall plant volume. This cell wall strengthening effect has also been validated in the drought resistance-related research on wheat by Boora et al. (2023b). Insufficient supply of low-concentration SiO_2_ fails to effectively activate osmotic adjustment and cell wall reinforcement, making it difficult to alleviate drought damage, resulting in a small difference in volume compared with the control group. As stated in existing studies on the concentration effect of silicon nanoparticles in drought resistance regulation, medium-concentration SiO_2_ tends to cause imbalance in plant nutrient distribution, interfere with normal water and physiological metabolism, and aggravate the impact of drought stress, thereby inhibiting seedling volume growth. This dual regulatory characteristic has also been reported in relevant studies on the application of silicon nanoparticles to cucumber. Although ultra-high-concentration silicon has a prominent growth-promoting effect, it tends to alter the cellular physicochemical environment in the long term and carries the risk of physiological burden.

**Figure 12:**
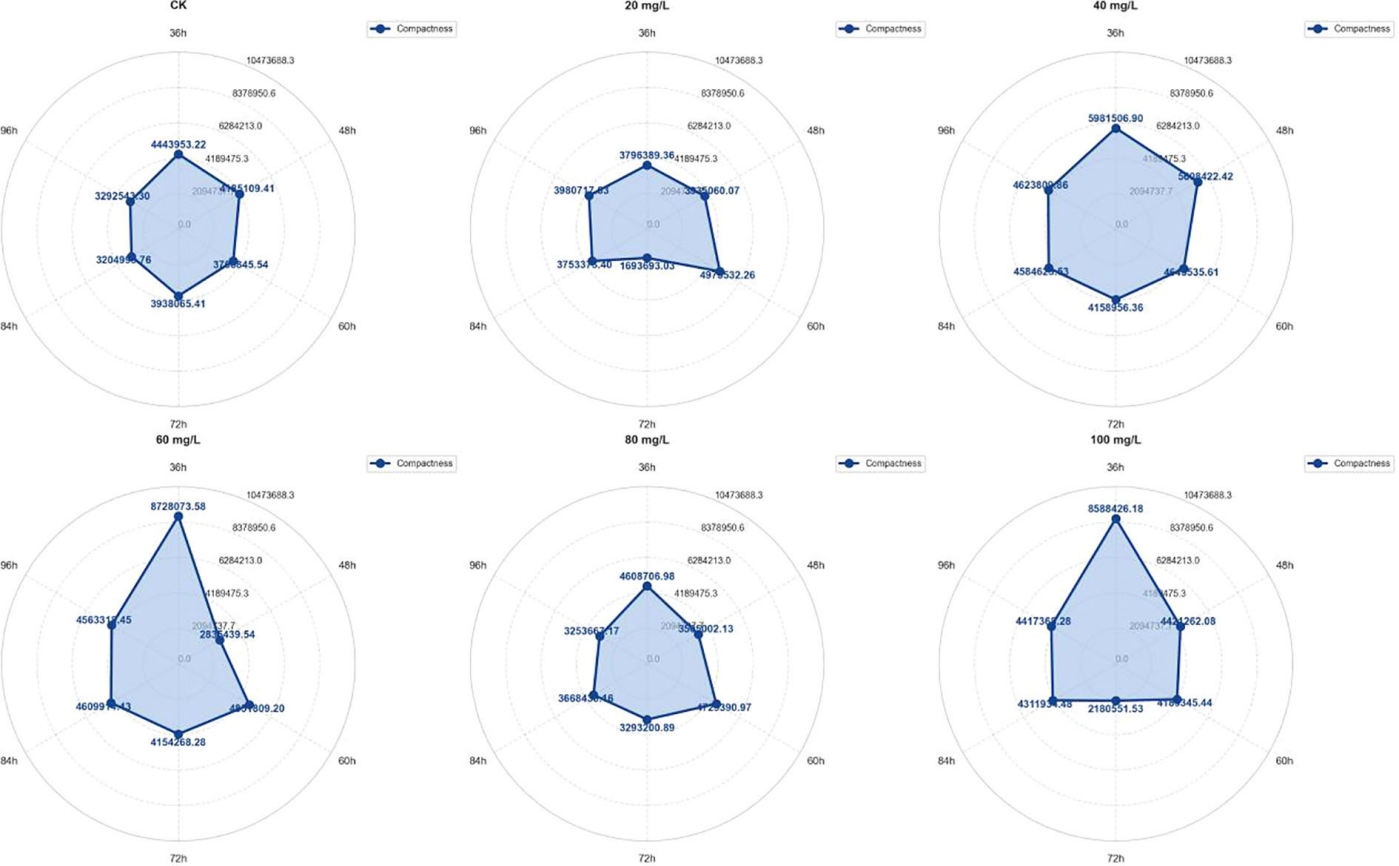
Dynamic change trend of compactness of cucumber seedlings primed with different concentrations of SiO_2_ nanoparticles (36h–96h).

As shown in **Figure 12**, under drought conditions, the compactness of cucumber seedlings in the CK control group (silicon concentration 0) remained stable overall during the whole treatment period, with moderate baseline values and no obvious drastic fluctuations. The compactness of the 20 mg/L SiO_2_ low-concentration treatment group was lower than that of the control group at all time points, being the lowest among all groups, showing a persistent characteristic of insufficient compactness. The compactness of the 40 mg/L treatment group increased significantly compared with 20 mg/L, rose gradually over time and peaked in the middle stage, but the overall improvement effect was limited. The 60 mg/L SiO_2_ treatment exhibited a sharp increase in compactness at the initial stress stage, significantly higher than all other treatments and reaching the maximum value for the whole period (Hu et al., 2023); it then decreased slowly with prolonged stress time. The 80 mg/L group showed relatively low overall compactness, only slightly higher than the weak 20 mg/L group. The 100 mg/L high-concentration treatment maintained high compactness at the initial stage and declined slowly later, but its overall compactness was still superior to the control and medium–low concentration groups. In summary, SiO_2_ regulation on cucumber seedling compactness shows significant concentration effects and time specificity: inhibited by low concentrations, slightly improved by medium concentrations, and significantly enhanced at the initial stage by medium–high concentrations. This concentration-dependent dynamic response pattern is highly consistent with the findings of Li et al. on silicon-regulated tissue compactness in cucumber seedlings (Li Y. et al., 2024).

According to the dedicated study by Li et al. on drought resistance of cucumber seedlings regulated by SiO_2_ nanoparticles, plant compactness mainly depends on cell wall mechanical strength, cell arrangement density, and water retention and structural stability under drought. Drought stress causes cell dehydration and shrinkage, loose tissue structure, and reduced seedling compactness. This mechanism underlying the effect of drought on plant tissue structure has been systematically confirmed in the study of Riaz et al. on drought responses of vegetable seedlings (Riaz et al., 2023). Exogenous SiO_2_ can form silicon deposition on the cell walls of epidermal cells, parenchyma, and vascular bundles, thickening cell walls and enhancing tissue hardness and compactness, thus improving compactness. This core regulatory pathway is fully consistent with the silicon-mediated cell wall strengthening mechanism reported by Li Y. et al. (2024). Combined with the dose-effect rule of nano-silicon reported by Riaz et al., a moderately high concentration of 60 mg/L silicon can rapidly trigger silicon transport and enrichment; massive silicide accumulation at the early stress stage reinforces plant structure, leading to a short-term surge in compactness. With continuous drought, excessive silicon deposition impedes intercellular substance transport, resulting in a late decline in compactness. The low concentration of 20 mg/L provides insufficient silicon to effectively complete cell wall silicification and reinforcement; coupled with the loosening effect of drought-induced water loss, compactness decreases significantly. Although the excessively high concentration of 100 mg/L ensures sufficient silicon deposition and high baseline compactness, it alters the cellular physicochemical microenvironment in the long term, imposes structural and metabolic loads, and causes compactness to decrease gradually over time.

**Figure 13:**
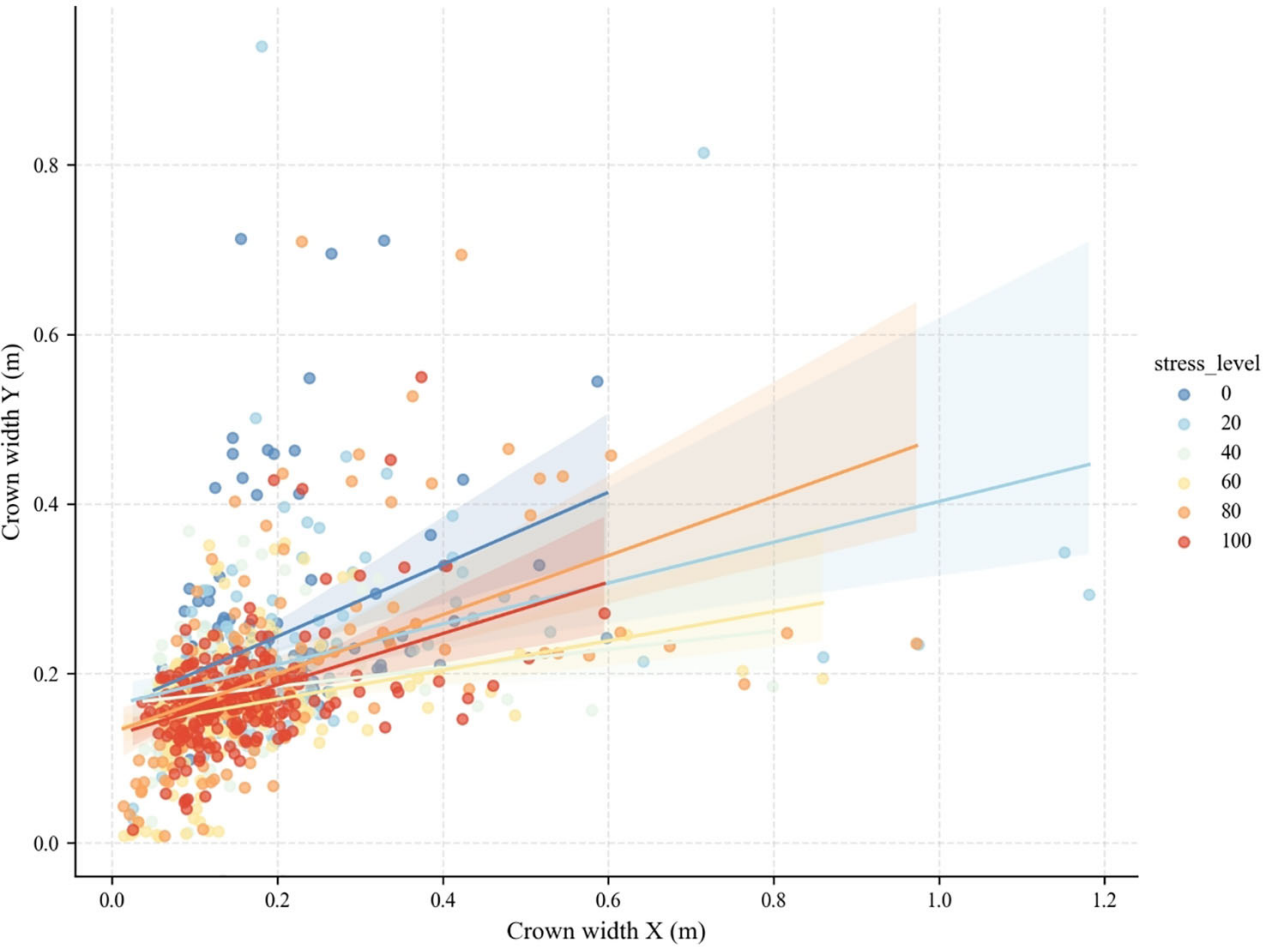
Distribution and variation of crown width (X/Y directions) of cucumber seedlings primed with different concentrations of SiO_2_ nanoparticles.

As shown in **Figure 13**, under drought stress, cucumber seedlings in the CK control group exhibited good coordination in lateral crown width growth, with concentrated spatial distribution and strong synchronization in bidirectional expansion. Under treatment with a low concentration of 20 mg/L SiO_2_, seedlings showed a wider crown width distribution range and greater lateral leaf expansion, and the crown expansion effect was superior to most other treatment groups. The 40 mg/L SiO_2_ treatment group had the smallest overall crown width, with dense point distribution and significantly inhibited lateral growth. As the SiO_2_ concentration increased to 80 mg/L, the synchronization of bidirectional crown width growth decreased, and crown uniformity declined. The 100 mg/L high-concentration SiO_2_ group had a high proportion of low crown width values, and overall lateral expansion was significantly restricted. Overall, low-concentration SiO_2_ is conducive to crown width expansion in cucumber seedlings, while medium and high concentrations inhibit crown development, disrupt the synchronization of bidirectional crown growth, and present obvious negative concentration effects (Boora et al., 2023b). This differential regulatory characteristic is highly consistent with the findings of Wang et al. on silicon-mediated canopy structure in cucumber (Wang et al., 2021).

According to the study by Wang et al. on silicon regulation of canopy development in cucumber under drought stress, crown width in cucumber seedlings depends on leaf expansion, lateral branch development, and photosynthate distribution. Drought reduces leaf cell turgor, inhibits epidermal cell elongation, and restricts crown expansion. This inhibitory mechanism of drought on canopy development has been widely validated in drought resistance studies of various vegetable crops (Riaz et al., 2023; Wang et al., 2021). Appropriately low-concentration SiO_2_ can improve the osmotic adjustment ability of plants, relieve drought-induced water loss, maintain normal leaf turgor, promote leaf flattening and lateral bud germination, and expand the crown width distribution range. Combined with the study by Guo et al. on the effects of biosynthesized silicon nanoparticles (Si NPs) at different concentrations (30 ppm, 60 ppm, 90 ppm, 120 ppm) on physiological responses and gene expression of wheat under drought stress (Boora et al., 2023b), medium and high concentrations of exogenous SiO_2_ alter the plant’s material distribution strategy, directing photosynthates preferentially to stem longitudinal growth and root development, reducing transport to leaves and aboveground lateral tissues and inhibiting lateral leaf development. Meanwhile, excessive silicon disturbs the polar growth balance of plants, weakens the synchronization of bidirectional crown growth, ultimately resulting in smaller crown width, compact plant architecture, and reduced uniformity.

### 3.3 Experimental summary

Drought stress significantly inhibits the normal growth of cucumber seedlings, resulting in restricted plant height elongation, suppressed plant expansion, loose tissue structure, inhibited crown width lateral development, and overall weak growth. Exogenous application of nano-SiO_2_ shows a significant concentration-dependent and trait-differentiated response in regulating various phenotypic indicators, and higher concentration does not lead to better effects (Rakesh et al., 2024).

Nano-SiO_2_ at a low concentration (20 mg/L) has limited ability to alleviate drought stress, showing no promotion effect on plant height and even growth fluctuation inhibition, with insignificant increase in plant volume and significantly reduced compactness. It only promotes leaf expansion and broadens the crown width distribution range to a certain extent, resulting in weak overall improvement effects. This limited regulatory effect of low-concentration silicon is fully consistent with the reported dose–response pattern of SiO_2_ nanoparticles in cucumber (Boora et al., 2023b; Rakesh et al., 2024).

Nano-SiO_2_ at medium concentrations (40–60 mg/L) exhibits trait bidirectional regulation characteristics. It can effectively deposit on cell walls, improve seedling tissue structure and compactness, and maintain stable plant height growth. However, this concentration changes photosynthate distribution, significantly inhibits plant volume enlargement and crown width lateral expansion, leading to compact plant architecture and restricted lateral growth of seedlings.

Nano-SiO_2_ at a moderately high concentration (80 mg/L) presents the best comprehensive stress resistance and growth promotion effects. It effectively relieves the inhibition of stem cell elongation caused by drought, significantly improves late plant height, steadily increases plant volume, and optimizes tissue structure compactness. Although it slightly reduces the bidirectional growth synergy of crown width, it coordinately improves various phenotypic formations and significantly alleviates seedling wilting and weak growth under drought (Lérida-Viso et al., 2023).

Nano-SiO_2_ at a high concentration (100 mg/L) exerts negative physiological effects. Although it greatly increases plant volume and maintains high compactness, it severely restricts crown width expansion, disturbs nutrient distribution balance, and shows lower plant height promotion than 80 mg/L. Long-term application tends to cause metabolic burden and is unfavorable to balanced development of overall morphology. According to the findings of Riaz et al., long-term application of nano-silicon at this concentration tends to impose a dual burden on plant ion metabolism and photosynthate partitioning, which is unfavorable to the balanced development of overall plant morphology.

In summary, nano-SiO_2_ can improve the phenotype of cucumber seedlings under drought by strengthening cell walls, enhancing water retention capacity, and regulating photosynthetic material distribution. 80 mg/L is the optimal application concentration to coordinate various morphological traits and alleviate drought damage. Low concentrations have insufficient enhancement effects, medium–high concentrations cause inhibition of single traits, and excessively high concentrations easily induce growth imbalance.

## 4 Conclusions

### 4.1 Contributions

Focusing on the core requirements of drought-stressed cultivation of protected cucumbers, this study conducted systematic research by integrating 3D point cloud technology, deep learning-based semantic segmentation, and plant stress resistance cultivation methods, aiming to address the key issues: the difficulty in accurate 3D phenotypic characterization of cucumber seedlings and the unclear regulation mechanism of SiO_2_ nanoparticles on drought stress.

At the phenotypic detection level, a compact and low-redundancy point cloud segmentation model DDCANet for cucumber seedlings was constructed by multi-dimensional optimization of the PointNet++-SSG model, embedding modules such as adaptive density awareness and channel attention normalization, and designing a composite loss function (Yan et al., 2024). This model successfully achieved high-precision segmentation of stem and leaf organs, as well as automated and non-destructive extraction of core 3D phenotypes including plant height, volume, compactness, and crown width. Evaluated via 3-fold cross-validation, the model attained a mean mIoU of 89.01 ± 0.32%, with its stem IoU showing a maximum improvement of 30.5% over the classic model. These stable and reproducible results effectively overcome the technical shortcomings of conventional manual measurement and 2D image analysis methods.

At the drought stress regulation level, seed soaking treatments with SiO_2_ nanoparticles at gradient concentrations of 0–100 mg/L were set, combined with 60 g/L PEG solution to simulate drought stress. The dynamic phenotypic changes of cucumber seedlings from 36 to 96 h after sowing were systematically monitored, and the concentration effect and time-dependent cumulative effect of the regulatory role of SiO_2_ nanoparticles on the growth of cucumber seedlings under PEG-simulated drought conditions were clarified. 80 mg/L was determined as the optimal application concentration, which could comprehensively improve key phenotypes such as plant height and volume of seedlings under drought stress. Low-concentration treatments showed weak effects, medium concentrations exhibited bidirectional regulation characteristics, and high concentrations presented obvious negative physiological effects.

This study revealed the growth regulatory effect of SiO_2_ nanoparticles on cucumber seedlings under drought stress from the perspective of phenotypic response and established an integrated research system of “accurate phenotypic detection – optimization of stress resistance regulation”. It not only provides clear concentration thresholds and theoretical–technical support for the scientific application of SiO_2_ nanoparticles in drought stress cultivation of protected cucumbers, but also offers a new approach for phenomics research on crop abiotic stress responses.

### 4.2 Limitations

Despite the above phased achievements, this study still has certain limitations due to constraints of research conditions, experimental duration, and technical approaches.

First, the dimension of phenotypic research is relatively limited. This study only focuses on morphological phenotypic parameters such as plant height and volume, without integrating physiological and biochemical indicators including photosynthetic rate and antioxidant enzyme activities. In addition, accurate characterization of the 3D root phenotype of cucumber seedlings has not been performed, making it impossible to conduct a multi-dimensional comprehensive analysis of “morphology–physiology, aboveground–underground” and difficult to further dissect the drought-resistant physiological mechanism of SiO_2_ nanoparticles (Wu et al., 2022).

Second, the generalization ability of the model is insufficient. The DDCANet model was constructed and trained only using samples of the single cucumber variety Trailing under artificial incubator conditions, without considering the influences of complex factors such as varying illumination, plant occlusion, and background noise under different cucumber varieties, natural greenhouse or field conditions. The applicability and robustness of the model in actual production scenarios still need to be verified. It is important to note that the limited size of the subset used in this study results in a small number of samples during testing. Consequently, segmentation errors in individual samples may introduce minor fluctuations in the evaluation metrics. Additionally, the conclusions presented here are validated only on the current dataset and have not yet been confirmed on larger independent external datasets.

Third, the depth of regulatory mechanism research is inadequate. This study only revealed the drought-resistant effect and dose-response pattern of SiO_2_ nanoparticles at the phenotypic level, without further exploring their absorption, translocation, and accumulation rules in cucumber seedlings, nor analyzing their regulatory effects on the expression of drought stress-related genes and endogenous hormone signaling networks at the molecular level. Furthermore, the experimental design lacked a control group under normal water conditions, as all treatments were conducted in a PEG-simulated drought environment. Consequently, it is impossible to fully distinguish the specific regulatory effect of SiO_2_ nanoparticles on drought stress from their general growth-regulating role, and the relevant conclusions need to be further verified under well-watered conditions.

Fourth, the experimental scenario and duration are limited. The experiment was only conducted in an artificially controlled environment with PEG-simulated drought, focusing solely on the seedling stage from 36 to 96 hours after sowing. The research period was not extended to the adult and fruiting stages, and verification under natural field drought conditions was lacking. Therefore, the persistence of the drought-resistant effect of SiO_2_ nanoparticles and their subsequent impacts on the late growth, yield formation, and fruit quality of cucumbers cannot be clarified.

### 4.3 Future prospects

In view of the limitations of this study and combined with the requirements for high-quality development of protected agriculture as well as the trends of crop phenomics and nano-agricultural technology, in-depth research will be carried out in various aspects to continuously improve the research system and enhance the theoretical and application value of the research results.

Firstly, a multi-dimensional phenotypic monitoring system will be established by integrating hyperspectral imaging, root scanning and other technologies. On the basis of accurately extracting morphological phenotypes, physiological and biochemical phenotypic parameters such as photosynthesis and antioxidant capacity will be obtained synchronously. Combined with multi-omics analysis including transcriptomics and metabolomics, the comprehensive mechanism underlying the growth regulation of cucumber seedlings by SiO_2_ nanoparticles under drought stress will be systematically analyzed from phenotypic response to molecular mechanism.

Secondly, the practicability and generalization ability of the DDCANet model will be further optimized. The point cloud dataset covering different cucumber varieties, growth stages and environmental conditions will be expanded for iterative optimization and lightweight compression of the model. The model will be deployed on embedded devices to develop a portable, high-throughput rapid 3D phenotyping system for cucumber seedlings, realizing the engineering application of the model. In future work, we will conduct full-scale independent validation using the complete reserved external dataset, which will further verify the statistical validity of our findings and the generalization performance of the proposed model.

Thirdly, the drought-stressed regulation mechanism of SiO_2_ nanoparticles will be deeply explored. By means of isotope tracing, electron microscopy scanning, quantitative real-time PCR and other technologies, the transport, distribution characteristics and action sites of SiO_2_ nanoparticles in cucumber seedlings will be clarified, and their regulatory effects on drought stress-related genes and signaling pathways will be analyzed, providing more in-depth theoretical support for the precise application of nano-agricultural technology. Meanwhile, Subsequent experiments will add a non-drought control group treated with SiO_2_ alone, so as to accurately distinguish the drought-specific alleviation effect of SiO_2_ nanoparticles from their basic growth regulatory effect, and further improve the chain of causal demonstration. And in parallel, multi-scenario and long-term verification experiments will be conducted under natural field drought conditions (Li et al., 2025) to track and monitor the effects of SiO_2_ nanoparticle treatment on the growth, yield and quality of cucumber at the adult plant stage. In addition, the synergistic regulation effects of SiO_2_ nanoparticles with other drought-resistant measures such as humic acid and trace elements will be investigated to screen the optimal composite regulation scheme.

Finally, the research and application scope will be extended. The optimized model will be adapted to the 3D phenotyping of other protected vegetable seedlings such as tomato and pepper to build a multi-crop universal phenotypic segmentation model. Furthermore, the optimal drought-stressed regulation scheme of SiO_2_ nanoparticles will be integrated with the intelligent environmental control and precise water-fertilizer management systems of protected agriculture to establish a precise drought-resistant cultivation system for protected cucumbers, promoting the deep integration of nano-agricultural technology and intelligent protected agriculture.

### 4.4 Summary

In summary, through the integration of technical innovation and cultivation experiments, this study successfully constructed a compact and low-redundancy 3D point cloud segmentation model suitable for cucumber seedlings, achieving accurate and non-destructive detection of drought-stressed phenotypes. Meanwhile, the optimal application concentration and phenotypic response pattern of SiO_2_ nanoparticles for phenotypic regulation of cucumber seedlings under PEG-simulated drought conditions were clarified, laying a theoretical and technical foundation for the application of nanotechnology in drought-stressed cultivation of protected cucumbers.

However, the research still has limitations in terms of phenotypic dimensions, model generalization, depth of mechanism analysis, and experimental scenarios. In the future, the research system will be continuously improved through multi-dimensional phenotype construction, model optimization, in-depth mechanism exploration, multi-scenario verification, and technical integration, so as to promote the transformation of research results from the laboratory to field production. This will provide more comprehensive support for the stress-resistant cultivation of protected vegetables and the industrial application of nano-agricultural technology, and boost the high-quality and high-efficiency development of protected agriculture.

## Data Availability

The raw data supporting the conclusions of this article will be made available by the authors, without undue reservation.
